# Antibody feedback regulates immune memory after SARS-CoV-2 mRNA vaccination

**DOI:** 10.1038/s41586-022-05609-w

**Published:** 2022-12-06

**Authors:** Dennis Schaefer-Babajew, Zijun Wang, Frauke Muecksch, Alice Cho, Maximilian Loewe, Melissa Cipolla, Raphael Raspe, Brianna Johnson, Marie Canis, Justin DaSilva, Victor Ramos, Martina Turroja, Katrina G. Millard, Fabian Schmidt, Leander Witte, Juan Dizon, Irina Shimeliovich, Kai-Hui Yao, Thiago Y. Oliveira, Anna Gazumyan, Christian Gaebler, Paul D. Bieniasz, Theodora Hatziioannou, Marina Caskey, Michel C. Nussenzweig

**Affiliations:** 1 Laboratory of Molecular Immunology, The Rockefeller University, New York, NY USA; 2 Laboratory of Retrovirology, The Rockefeller University, New York, NY USA; 3 Howard Hughes Medical Institute, New York, NY USA

**Keywords:** RNA vaccines, Antibodies, Immunological memory, Germinal centres

## Abstract

Feedback inhibition of humoral immunity by antibodies was first documented in 1909^1^. Subsequent studies showed that, depending on the context, antibodies can enhance or inhibit immune responses^2,3^. However, little is known about how pre-existing antibodies influence the development of memory B cells. Here we examined the memory B cell response in individuals who received two high-affinity anti-SARS-CoV-2 monoclonal antibodies and subsequently two doses of an mRNA vaccine^4–8^. We found that the recipients of the monoclonal antibodies produced antigen-binding and neutralizing titres that were only fractionally lower compared than in control individuals. However, the memory B cells of the individuals who received the monoclonal antibodies differed from those of control individuals in that they predominantly expressed low-affinity IgM antibodies that carried small numbers of somatic mutations and showed altered receptor binding domain (RBD) target specificity, consistent with epitope masking. Moreover, only 1 out of 77 anti-RBD memory antibodies tested neutralized the virus. The mechanism underlying these findings was examined in experiments in mice that showed that germinal centres formed in the presence of the same antibodies were dominated by low-affinity B cells. Our results indicate that pre-existing high-affinity antibodies bias germinal centre and memory B cell selection through two distinct mechanisms: (1) by lowering the activation threshold for B cells, thereby permitting abundant lower-affinity clones to participate in the immune response; and (2) through direct masking of their cognate epitopes. This may in part explain the shifting target profile of memory antibodies elicited by booster vaccinations^9^.

## Abstract (web summary)

Pre-existing high-affinity antibodies alter germinal centre and memory B cell selection by lowering the activation threshold for B cells and through direct masking of their cognate epitopes, thereby permitting a diverse set of abundant lower-affinity clones targeting alternate epitopes to participate in the immune response.

## Main

**Fig. 1:**
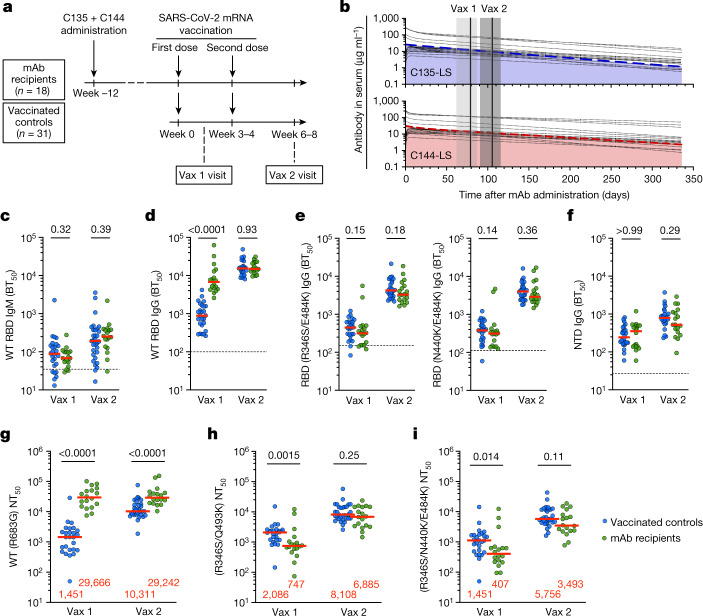
Study design and plasma antibody activity. **a**, Schematic of the study design, with markers denoting weeks relative to the time of the first vaccine dose. mAb, monoclonal antibody. **b**, Serum levels of C135-LS (top, blue) and C144-LS (bottom, red) over time are shown. The thick coloured dashed lines indicate the median serum concentrations among monoclonal antibody recipients (*n* = 18), and the thin dotted black lines represent individual participants. The two solid vertical lines indicate the median value and the grey shaded areas indicate the range of time from monoclonal antibody administration to vaccination. **c**–**f**, The half-maximal plasma binding titre (BT_50_) to RBD after one (vax 1) and two doses (vax 2) of mRNA vaccination for monoclonal antibody recipients (*n* = 18, green) and controls (*n* = 26, blue). Each dot represents one individual. The dashed horizontal lines represent the median binding activity of pre-pandemic plasma samples from healthy individuals that were used as negative controls. **c**,**d**, IgM (**c**) and IgG (**d**) binding titres to WT RBD. **e**, IgG binding to R346S/E484K (left) and N440K/E484K RBDs (Extended Data Fig. 1). **f**, IgG binding to the NTD. **g**–**i**, Plasma half-maximal neutralizing titre (NT_50_) values for monoclonal antibody recipients (*n* = 18, green) and controls (*n* = 26, blue) against HIV-1 pseudotyped with SARS-CoV-2 WT S (**g**), R346S/Q493K mutant S (**h**) and R346S/N440K/E484K mutant S (**i**) (Extended Data Fig. 2). The S protein in the pseudoviruses in **g**–**i** contained an R683G substitution. The red horizontal bars in **c**–**i** and the red numbers in **g**–**i** represent the median values. Statistical significance in **c**–**i** was determined using two-tailed Mann–Whitney *U*-tests comparing the differences between monoclonal antibody recipients and controls for each timepoint independently; *P* values are shown above the plots. All of the experiments were performed at least in duplicate.

To examine how passive administration of monoclonal antibodies might influence subsequent humoral responses to vaccination in humans, we studied a group of 18 healthy volunteers who received a single dose of a combination of two long-acting monoclonal antibodies against SARS-CoV-2 and subsequently received two doses of a SARS-CoV-2 mRNA vaccine (Fig. 1a). The 2 antibodies—C144-LS and C135-LS—bind to class 2 and 3 epitopes on the RBD of the SARS-CoV-2 spike (S) protein with high affinity (dissociation constant (*K*_d_) = 18 nM and *K*_d_ = 6 nM, respectively) and neutralize the virus with half-maximal inhibitory concentration (IC_50_) values of 2.55 and 2.98 ng ml^−1^, respectively^5,8^.

Between 13 January and 3 March 2021, 23 SARS-CoV-2-naive individuals received C144-LS and C135-LS (*n* = 21) or placebo (*n* = 2) in a first-in-human phase 1 clinical trial at the Rockefeller University Hospital (ClinicalTrials.gov: NCT04700163). The antibodies were modified to extend their half-life by introducing the M428L and N343S mutations into their Fc domains^10^ (LS). Individuals received a single dose of C144-LS and C135-LS IgG1 antibodies at a 1:1 ratio, starting with 100 mg of each subcutaneously (s.c.) in the lowest-dose group and up to 15 mg per kg intravenously (i.v.) in the highest-dose group. The participants were followed longitudinally to assess the safety and tolerability of the infused monoclonal antibodies and determine their pharmacokinetic properties.

A total of 18 out of the 21 phase 1 study participants who had received the monoclonal antibodies elected to receive SARS-CoV-2 mRNA vaccination and volunteered to enrol in a parallel observational study assessing their immune responses to SARS-CoV-2 vaccination (Supplementary Tables 1 and 2). The first and second vaccine doses were administered a median of 82 (range, 42–110) and 103 (range, 70–131) days after antibody administration (Supplementary Tables 1 and 2). At the time of vaccination, the plasma levels of C144-LS and C135-LS were between 5 and 100 µg ml^−1^ depending on the dosing group (Fig. 1b). The estimated half-lives of C144-LS and C135-LS were 69–99 days and 73–95 days, respectively.

The 18 vaccinated antibody recipients were compared with a cohort of 31 randomly selected mRNA vaccine recipients with no previous history of infection^9,11^ (Fig. 1a and Supplementary Table 1). Both of the groups were sampled 13–28 (median, 19) and 15–91 (median, 29) days after their first and second vaccine doses, respectively. The two cohorts were relatively matched for demographic characteristics and vaccine formulation (Supplementary Tables 1 and 2), and none of the individuals included in the study seroconverted to nucleocapsid (N) at any time during the observation period, suggesting that they remained infection naive.

## Plasma antibody reactivity

**Extended Data Fig. 1:**
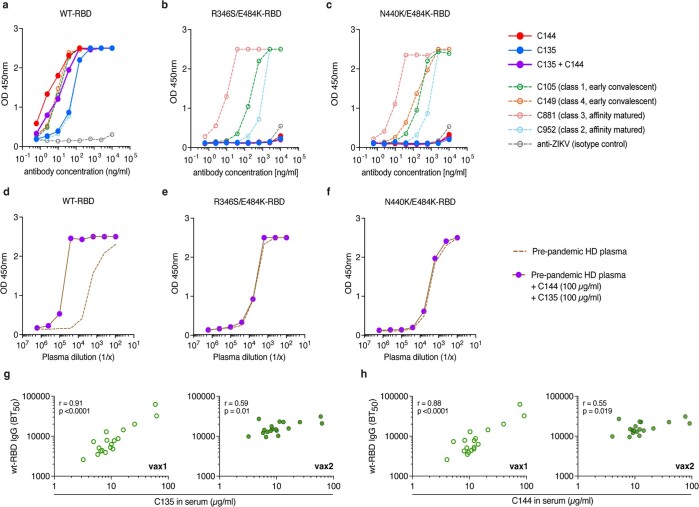
C135 and C144 – selectively abrogated binding to mutant RBDs and correlations with plasma reactivity. **a**-**f**, Monoclonal antibody binding to mutant forms of RBD. **a**-**c**, Graphs show concentration-dependent antibody binding to (**a**) WT, (**b**) R436S/E484K, and (**c**) N440K/E484K RBDs by C144, C135, and Class 1 (C105), Class 2 (C952), Class 3 (C881), and Class 4 (C149)^5,6,8,26^. **d**-**f**, Graphs show concentration dependent pre-pandemic healthy donor plasma binding to (**d**) WT, (**e**) R436S/E484K, and (**e**) N440K/E484K RBDs in the presence (purple) or absence (dotted lines) of 100 mg ml^−1^ of C135 and C144. Addition of C144 and C135 to plasma increases the binding activity of plasma against the WT but not the 2 mutant RBDs. **g**-**h**, Panels show the correlation of C135-LS (**g**) and C144-LS (**h**) serum levels at day 84 post-administration (around the times of vaccination as seen in Fig. 1b) with the total anti-WT RBD IgG antibody titre of mAb recipients (n = 18) after one (empty green circles) and two doses (solid green dots) of mRNA vaccination. Statistical significance in **g** (r = 0.9097 and r = 0.5891 with p < 0.0001 and p = 0.0101 for vax1 and vax2, respectively) and **h** (r = 0.8772 and r = 0.5483 with p < 0.0001 and p = 0.0185 for vax1 and vax2, respectively) was determined using the two-tailed Pearson correlation statistic. All experiments were performed at least in duplicate.

Plasma IgM and IgG antibody-binding activity against RBD were measured with an enzyme-linked immunosorbent assay (ELISA) using Wuhan-Hu-1 (wild type (WT)) and mutant forms of RBD (R346S/E484K and N440K/E484K) that eliminate binding by C144 and C135 but not class 1 or 4, or some affinity-matured class 2 or 3 antibodies^9,12^ (Extended Data Fig. 1a–f). When measured for WT RBD binding after one or two vaccine doses, the IgM titres of the monoclonal antibody recipients were not significantly different compared with the controls (Fig. 1c). By contrast, IgG anti-WT-RBD titres were significantly higher in monoclonal antibody recipients compared with in the controls after one vaccine dose, but equalized after the second dose (Fig. 1d; *P* < 0.0001 and *P* = 0.93, respectively). The initial difference was attributed to the infused monoclonal antibodies because, when the same samples were tested against either R346S/E484K or N440K/E484K mutant RBDs that interfere with C144 and C135 binding, plasma IgG antibody levels in the monoclonal antibody recipient samples were slightly, albeit not significantly, lower than the controls (Fig. 1e). By contrast, the relative contribution of endogenous anti-RBD antibodies increased over time, as illustrated by a decrease in the correlation between the monoclonal antibody serum levels and plasma binding (Extended Data Fig. 1g,h).

To determine whether the pre-existing antibodies against the RBD interfered with humoral immunity to independent domains of the SARS-CoV-2 S protein, the same plasma samples were tested for binding to the N-terminal domain (NTD). IgG titres to the NTD were similar in the monoclonal antibody recipients and controls (Fig. 1f). We conclude that high circulating levels of C144-LS and C135-LS do not interfere with IgM anti-RBD antibody responses and have only a small effect on IgG responses. Thus, the infused antibodies do not clear the vaccine antigen or measurably interfere with its overall ability to produce an immune response^13^.

## Neutralization

**Extended Data Fig. 2:**
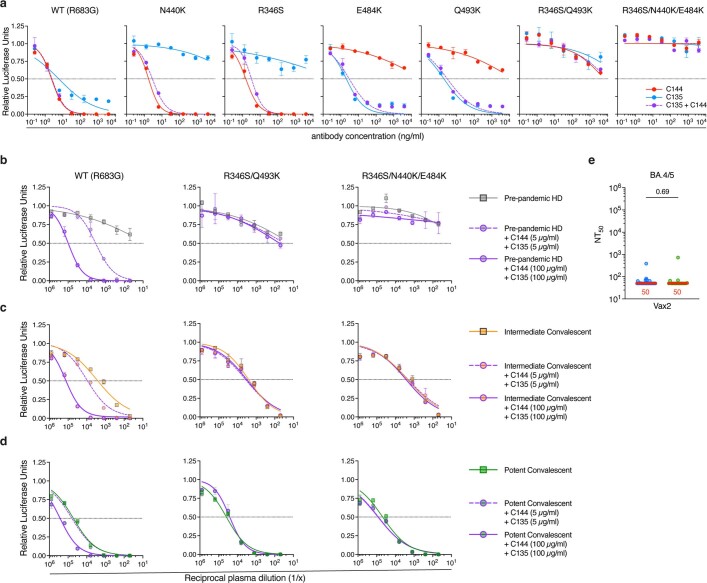
SARS-CoV-2 R346S/Q493K and R346S/N440K/E484K pseudotype virus neutralization by C135-LS and C144-LS, and BA.4/5 pseudotype neutralization by plasma. **a**, Graphs show concentration-dependent neutralization curves for SARS-CoV-2 pseudoviruses by monoclonal antibodies. C144 (red), C135 (blue), and their equimolar combination (purple). **b**, Pre-pandemic plasma (squares) neutralization of WT or R346S/Q493K or R346S/N440K/E484K pseudoviruses in the absence or presence of 5 (purple dashed circles) or 100 µg ml^−1^ (purple solid circles) of C135 and C144^14^. **c**-**d**, As in (**b**) but for convalescent plasma with intermediate (**c**, COV157)^12^ or strong (**d**, COV31)^12^ neutralizing activity. The horizontal lines in all panels indicate half-maximal neutralization. **e**, Plasma half-maximal neutralizing titre (NT50s) against HIV-1 pseudotyped with the BA.4/5 S^38^. Each dot represents one individual from the control group (n = 31, blue) or from the mAb recipient group (n = 18, green). Red horizontal bars and red numbers represent median values. Statistical significance was determined using the two-tailed Mann-Whitney. For **a**-**d**, individual symbols represent the mean of two independent experiments and error bars the standard deviation. All experiments were performed at least in duplicate.

To assess plasma neutralizing activity, we used HIV-1 pseudotyped with WT or mutant S proteins carrying a mutation in the furin-cleavage site (R683G)^14^. As expected, on the basis of the amount of C144-LS and C135-LS in circulation, neutralizing titres against the WT were significantly higher in monoclonal antibody recipients compared with in the controls at both timepoints (Fig. 1g; *P* < 0.0001). To determine the contribution of the endogenous neutralizing response to epitopes outside of the C144-LS and C135-LS target sites, we used viruses pseudotyped with S proteins containing the R346S/Q493K and R346S/N440K/E484K mutations that abolish the neutralizing activity of the two infused monoclonal antibodies (Extended Data Fig. 2a–d). Despite the initial dominance of class 1–2 epitopes among neutralizing antibodies elicited by mRNA vaccination^6^, the neutralizing titre of the control plasmas against the two mutant pseudoviruses were comparable to those against the WT (Fig. 1h,i), suggesting that a significant proportion of circulating endogenous neutralizing antibodies are unaffected by the R346S/Q493K and R346S/N440K/E484K mutations. After the first vaccine dose, monoclonal antibody recipients showed significantly lower neutralizing titres against the mutant pseudoviruses compared with the controls (2.7-fold and 3.5-fold for R346S/Q493K and R346S/N440K/E484K; Fig. 1h,i; *P* = 0.0015 and *P* = 0.014, respectively). Consistent with a recent report^13^, neutralizing activity improved and was no longer significantly different compared with the controls after the second vaccine dose (Fig. 1h,i). Similarly, we saw no discernible difference in plasma neutralization of the antigenically divergent BA.4/5 variant, which showed equal levels of immune evasion to the plasma antibodies in both groups (Extended Data Fig. 2e). In conclusion, recipients of C144-LS and C135-LS had high initial levels of serum neutralizing activity due to the passively administered antibodies and they developed their own neutralizing antibodies that were not sensitive to RBD mutations in the C144/C135 target sites after mRNA vaccination.

## Memory B cells

**Fig. 2:**
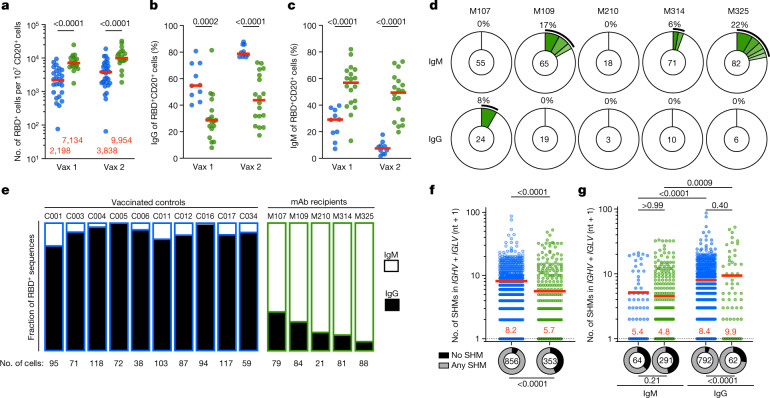
Anti-SARS-CoV-2 RBD memory B cells from vaccinated monoclonal antibody recipients. **a**–**c**, Flow cytometry enumeration and surface immunoglobulin expression of SARS-CoV-2 RBD-specific memory B cells after vax 1 and vax 2, isolated from monoclonal antibody recipients (green, *n* = 18) and control individuals^9,11^ (blue). *n* = 26 for vax 1 and *n* = 31 for vax 2 (**a**), and *n* = 10 (**b** and **c**). Each dot represents one individual. The red horizontal bars (and the numbers in **a**) show the median values. **a**, The number of WT RBD-specific memory B cells per 10 million CD20^+^ B cells (Extended Data Fig. 3a,b). **b**,**c**, The percentage of cells among WT RBD-binding CD20^+^ B cells that express cell surface IgG (**b**) or IgM (**c**). **d**, The distribution of antibody sequences derived from cells isolated from five vaccinated monoclonal antibody recipients after vax 2 (Extended Data Fig. 4a–d). The numbers in the inner circles indicate the number of sequences analysed for the respective individual. The green coloured slices indicate clonally expanded cells (same *IGHV* and *IGLV* genes, with highly similar CDR3s) within an individual. Slice size is proportional to the number of clonally related sequences, with the fraction of clonally expanded sequences summarized as a percentage (black outline). The white areas of the pie chart indicate the proportion of sequences that were isolated only once. **e**, The fraction of cells containing IgG (black) versus IgM (white) transcripts per individual (see also Extended Data Fig. 4e and refs. ^9,11^). **f**,**g**, Somatic hypermutation (SHM) shown as combined heavy- and light-chain variable region nucleotide (nt) substitutions plus one (*IGVH* + *IGVL* + 1), with each dot representing one sequence from monoclonal antibody recipients (green) or controls (blue). The ring plots at the bottom show the fraction of sequences with no (*IGVH* + *IGVL* + 1 = 1) versus any (*IGVH* + *IGVL* + 1 > 1) somatic hypermutation, and the encircled numbers indicate the number of sequences analysed for all cells irrespective of isotype (**f**), and for IgM and IgG analysed independently (**g**). The red horizontal bars and numbers in **f** and **g** indicate the mean values. Statistical significance was determined using two-tailed Mann–Whitney *U*-tests (**a**–**c** and **f**), Kruskal–Wallis tests with subsequent Dunn’s correction for multiple comparisons (**g**) and two-sided Fisher’s exact tests to compare fractions (**f** and **g**).

**Extended Data Fig. 3:**
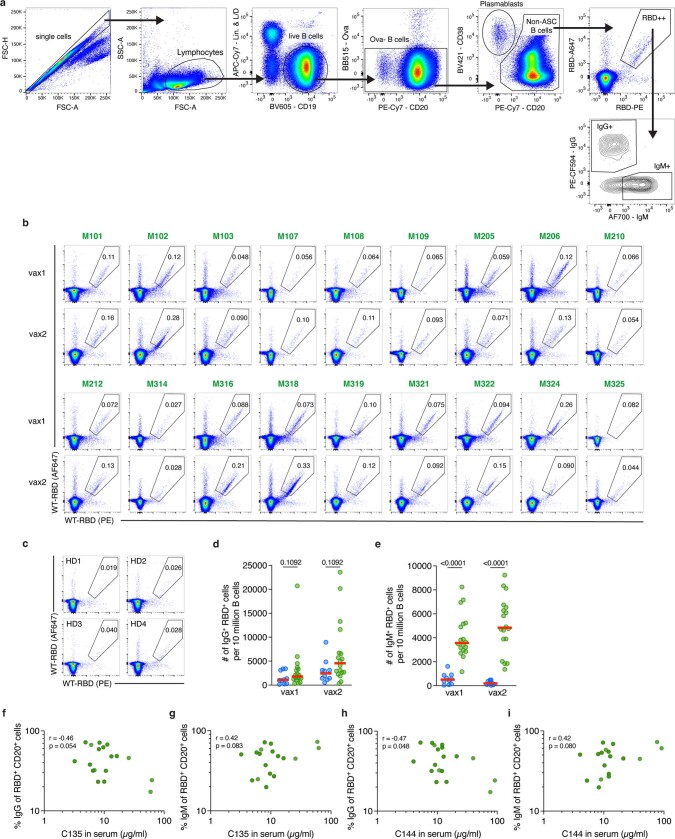
Flow-cytometry of human anti-RBD memory B cells. **a**, Gating strategy for flow-cytometry phenotyping. Gating was on single lymphocytes that were CD19^+^ and CD20^+^, and CD3^−^ CD8^−^ CD16^−^ Ova^−^ without uptake of live-dead dye (L/D). Antigen-specific cells were those with dual binding to Wuhan-Hu1 RBD-PE and RBD-AF647. Anti-IgG, -IgM were used to phenotype dual RBD-labelled B cells. **b**,**c**, Representative flow-cytometry plots of Wuhan Hu-1 RBD-binding memory B cells from mAb recipients after one and two doses of vaccination (**b**) and pre-pandemic health donors (**c**) serving as negative controls. Numbers in RBD-gate denote percentage of RBD dual-labelled cells of parent gate (see **a**). Corresponding flow-cytometry plots and gating strategy for vaccinated controls can be found in^11^. **d**-**e**, Number of IgG- (**d**) and IgM-expressing (**e**) WT RBD-specific memory B cells per 10 million CD20^+^ B cells. Each dot represents one individual from the mAb recipient (green, n = 18) and control group (blue, n = 10)^9,11^. Horizontal red bars denote median values. **f**-**i**, Panels show the correlation of C135-LS (**f**, **g**) and C144-LS (**h**,**i**) serum levels at day 84 post-administration (around the times of vaccination as seen in Fig. 1b) with the percentage of WT RBD-specific memory B cells expressing either IgG (**f**, **h**) or IgM (**g**, **i**) after two vaccine doses as assessed by flow-cytometry. Green dots represent individual mAb recipients (n = 18). Statistical significance was determined using the two-tailed Mann-Whitney for **d** and **e**, and the Pearson r correlation statistic was used for **f**-**i**.

In addition to the plasma cells that produce circulating antibodies, vaccination also elicits memory B cells that contribute to protection after re-exposure to the pathogen. These two cell types are selected by different mechanisms and, as a result, the antibodies that they produce show differing levels of affinity to the immunogen^15–17^. Although the feedback effects of antibodies on humoral responses have been investigated extensively, beginning in 1909^1,2^, little is known about their effects on the development of memory B cells. To investigate the effects of passive monoclonal antibody administration on B cell memory responses in humans, we used flow cytometry to enumerate and purify circulating memory B cells binding to phycoerythrin (PE)- and Alexa-Fluor-647 (AF647)-labelled RBDs^5^ (Extended Data Fig. 3a–c). mRNA vaccination elicited robust RBD-specific memory B cell responses in monoclonal antibody recipients that were approximately fourfold and threefold higher than in the controls after the first and second vaccine doses, respectively (Fig. 2a; *P* < 0.0001 and *P* < 0.0001, respectively). Thus, administration of C144-LS and C135-LS increases the magnitude of the anti-RBD memory B cell response compared with the controls.

Human memory B cells represent a diverse pool of cells that can develop in germinal centres or through an extrafollicular germinal-centre-independent pathway^18–20^. Memory B cells expressing class-switched and highly somatically mutated antibodies are primarily of germinal centre origin^19,21^. IgM-expressing memory B cells, which express antibodies that carry only small numbers of mutations, typically develop through a germinal-centre-independent pathway^22–25^. In control individuals, IgG-expressing RBD-specific memory cells comprised the majority of the memory B cell pool at both timepoints assayed. Consistent with the overall increase in anti-RBD memory cells, the absolute number of IgG-expressing cells was fractionally increased in monoclonal antibody recipients (Extended Data Fig. 3d). However, their relative contribution was significantly reduced at both timepoints, making up only 30% and 45% of the RBD-specific cells in monoclonal antibody recipients after one and two doses, respectively (Fig. 2b; *P* = 0.0002 and *P* < 0.0001). Consistent with the relative decrease in IgG^+^ memory B cells, more than half (57%) of the RBD-specific memory B cells from monoclonal antibody recipients were positive for cell-surface IgM after the first vaccine dose and this decreased only slightly to 49% after the second vaccine dose. By contrast, few such cells were found in the control group at that time (Fig. 2c and Extended Data Fig. 3e; all *P* < 0.0001). The skewed isotype ratio was correlated with the C144 serum concentration at the time of immunization (Extended Data Fig. 3f–i). We conclude that pre-existing high-affinity anti-RBD antibodies alter the immune response to SARS-CoV-2 mRNA vaccination to favour the development of IgM-expressing memory B cells.

## Memory B cell antibodies

**Extended Data Fig. 4:**
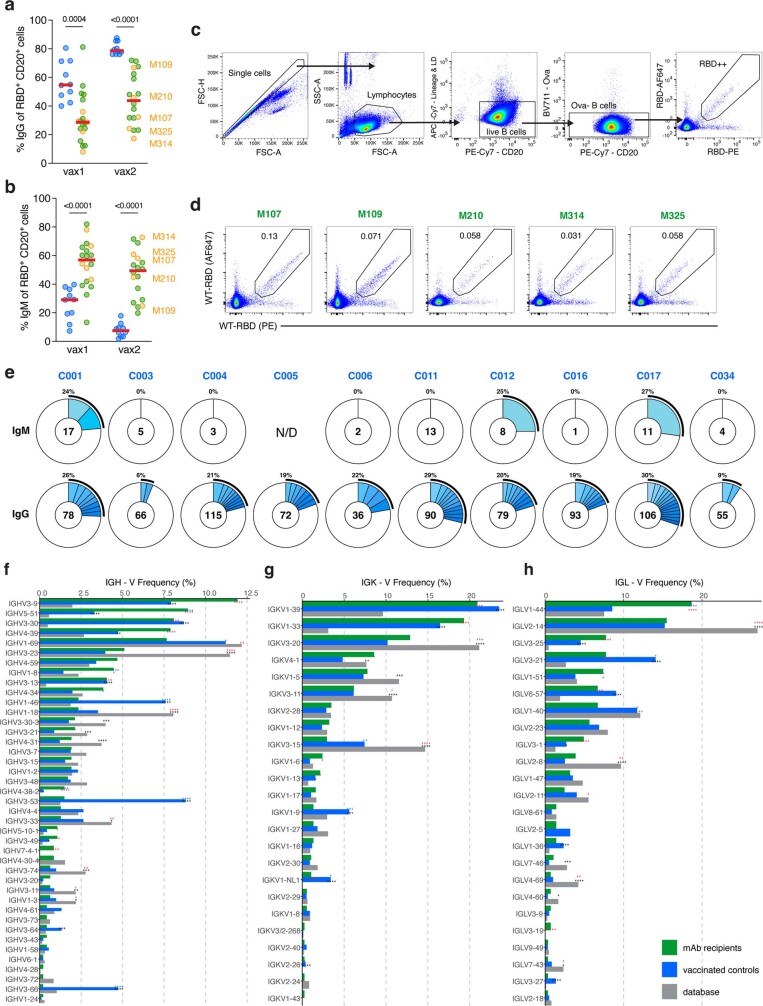
Fluorescence-activated cell sorting (FACS) of human anti-RBD memory B cells and subsequent BCR sequencing. **a**-**b**, Panels showing IgG (**a**) and IgM (**b**) surface expression of anti-RBD memory cells exactly as in Fig. 2b and c, but with the 5 representative individuals from whom cells were subsequently sorted highlighted in yellow. **c**, Gating strategy for single-cell sorting of RBD-specific memory B cells. Dual-labelled (RBD-PE^+^/-AF647^+^) CD20^+^ CD3^−^ CD8^−^ CD16^−^ Ova^−^ cells were sorted. **d**, Representative flow cytometry plots show RBD-binding cells that were sorted from the 5 mAb recipients. **e**, Pie charts show the distribution of antibody sequences derived from cells isolated from 10 vaccinated control individual after vax2. The upper panel shows IgM, and the lower panel depicts IgG sequences^9,11^. The number in the inner circle indicates the number of sequences analysed for the individual denoted above the circle. Slices coloured in shades of blue indicate cells that are clonally expanded (same IGHV and IGLV genes, with highly similar CDR3s). Pie slice size is proportional to the number of clonally related sequences. The black outline and % value indicate the frequency of clonally expanded sequences detected within an individual. White pie areas indicate the proportion of sequences isolated only once. For C005, there were no IgM transcripts amplified at the timepoint assayed. **f**-**h**, Comparison of the frequency distribution of V gene usage for the IgH and IgL among antibodies isolated from mRNA-vaccinated mAb recipients (this study) and controls^9,11^, after vax2, and from database of shared clonotypes of human antibodies from Soto et al.^43^. Graphs show relative abundance of human IGHV (**f**), IGKV (**g**), and IGLV (**h**) genes within the human V gene database (in grey, Sequence Read Archive accession SRP010970), antibodies isolated from mAb recipients (in green) or vaccinated controls (in blue). Colours of stars indicate levels of statistical significance for the following frequency comparisons: black – vaccinated controls vs. database; red – mAb recipients vs. database; blue – mAb recipients vs. vaccinated controls.

To gain further insights into the effects of pre-existing antibodies on the human memory response to SARS-CoV-2 mRNA vaccination, we purified RBD-specific memory B cells from five representative monoclonal antibody recipients after the second vaccine dose (Extended Data Fig. 4a,b). A total of 353 and 856 paired antibody sequences from monoclonal antibody recipients and previously characterized controls were examined, respectively^11^ (Fig. 2d, Extended Data Fig. 4c–e and Supplementary Table 3). IgM transcripts accounted for 70–94% of sequences recovered from monoclonal antibody recipients with an average of 9% belonging to expanded clones (Fig. 2d (top) and  2e). By contrast, IgG transcripts accounted for more than 90% of the immunoglobulin sequences isolated from the controls (Fig. 2e and Extended Data Fig. 4e). The relative IgM enrichment was more pronounced by the more sensitive PCR assay and may include cells that no longer express IgM on their surface. IgM memory cells originating from the extrafollicular non-germinal-centre pathway are generally less somatically mutated than IgG memory cells because they undergo fewer divisions^19,25^. Consistent with this idea, and the reversed ratio of IgM to IgG memory B cells in monoclonal antibody recipients, the antibodies obtained from these individuals showed significantly fewer somatic mutations compared with the controls (Fig. 2f; *P* < 0.0001). However, when comparing IgM or IgG cells independently, the average mutational burden was not significantly different between the monoclonal antibody recipients and the controls (Fig. 2g; *P* > 0.99 and *P* = 0.40 for IgM and IgG, respectively). Thus, IgM- and IgG-expressing B cells in vaccinated individuals who had received C144-LS and C135-LS carry normal numbers of somatic mutations, but the relative ratio of the two memory cell types is reversed, which accounts for the overall lower level of mutation in their memory compartment. Finally, in contrast to the controls, there was no enrichment for the VH3-53, VH1-69, VH1-46 and VH3-66 heavy chains, which often target class 1 and 2 epitopes. Instead, there was relative enrichment for the *VH3-9*, *VH5-51*, *VH4-39* and *VH1-8* genes (Extended Data Fig. 4f). The limited number of cells sequenced precludes definitive conclusions about the precise clonotype distribution in this population, but the relative change in *VH* gene use frequency implies that B cell recruitment into the memory compartment of monoclonal antibody recipients is altered. In summary, the data suggest that pre-existing antibodies can alter the cellular and molecular composition of the RBD-specific MBC compartment that develops in response to mRNA vaccination.

**Fig. 3:**
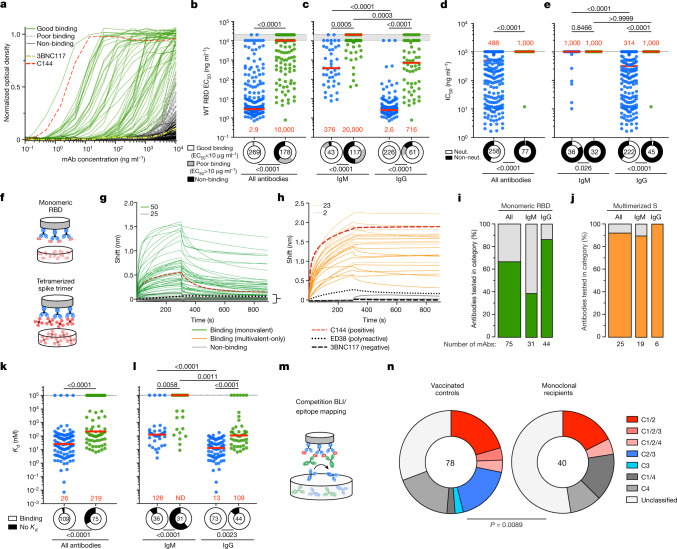
Anti-SARS-CoV-2 RBD memory antibodies from vaccinated monoclonal antibody recipients. **a**–**c**, Monoclonal antibody binding to WT RBD. **a**, ELISA binding of monoclonal memory antibodies derived from monoclonal antibody recipients is shown. Each curve represents one antibody. The green curves show EC_50_ values of <10 µg ml^−1^; the grey dashed lines show EC_50_ values of >10 µg ml^−1^; the solid black lines are antibodies that were below or equal to the negative control anti-HIV1 antibody 3BNC117 (thick yellow dashed line). C144 (thick, red dashed line) was used as a positive control. **b**, EC_50_ values derived from **a** for monoclonal antibody recipients (green) and controls (blue) for all antibodies, irrespective of isotype. **c**, EC_50_ values as in **b**, but IgM and IgG were analysed independently. The grey shaded area between the horizontal dotted lines indicates antibodies with EC_50_ > 10 µg ml^−1^ (poor binding) and non-binding antibodies, arbitrarily grouped at 10 and 20 µg ml^−1^, respectively. The ring plots summarize the fraction of all antibodies tested for the respective groups (encircled number). **d**, IC_50_ values for all monoclonal antibodies isolated from vaccinated monoclonal antibody recipients (green) or control individuals (blue). The ring plots illustrate the fraction of non-neutralizing (non-neut.; IC_50_ > 1,000 ng ml^−1^) antibodies (black slices) among all antibodies tested for the respective group (encircled number). **e**, IC_50_ values as described in **d**, but IgM and IgG antibodies were analysed independently. **f**–**l**, Monoclonal antibody binding to monomeric and multimerized antigen by BLI. **f**, Schematic of monomeric binding measurements in which IgG was immobilized onto the biosensor chip and subsequently exposed to monomeric RBD (top), and multimeric binding using 6P-stabilized WT SARS-CoV-2 S protein trimers that had been tetramerized using streptavidin (bottom). **g**, BLI traces obtained under monovalent conditions as shown in **f** (top). Each curve represents one antibody. The coloured solid lines denote binding above the background represented by polyreactive antibody ED38^35^ (dotted black line) and anti-HIV-1 antibody 3BNC117 (dashed black line). The grey lines show non-binding antibodies. C144 (thick, red dashed line) was used as a positive control. **h**, BLI traces as described in **g** for antibodies that showed no measurable binding in **g** and were subsequently tested for binding under polyvalent conditions as illustrated in **f** (bottom). **i**, The percentage of binding antibodies under monovalent conditions for all antibodies and by isotype. The values below the bars indicate the number of antibodies tested. **j**, The percentage of binding antibodies as described in **i** for the antibodies shown in **h**. **k**, *K*_d_ values derived under monomeric binding conditions in **g** for monoclonal antibody recipients (green) and controls (blue) irrespective of isotype. The ring plots illustrate the fraction of antibodies tested for the respective group (encircled number) that measurably bound to monomeric RBD (binding, white) and those for which a *K*_d_ value could not be established (no *K*_d_, black). **l**, *K*_d_ values as described in **k** were analysed independently for IgM and IgG. **m**, Schematic of the BLI competition experiment: (1) the capture antibody of known epitope specificity (class-reference antibody) was bound to the biosensor chip; (2) exposed to antigen; and (3) the antibody of interest was added to the chip. **n**, The distribution of the epitopes targeted. The number in the centre is the number of antibodies tested. Slices coloured in shades of red and blue represent class 1, 2 and 3 or combined epitopes, and shades of grey represent class-4-containing epitopes or epitopes that could not be classified. For **b**–**e**, **k** and **l**, the red horizontal bars and numbers represent the median values. ND, not determined. Statistical significance was determined using two-tailed Mann–Whitney *U*-tests (**b**, **d** and **k**), Kruskal–Wallis tests with subsequent Dunn’s correction for multiple comparisons (**c**, **e** and **l**), two-sided Fisher’s exact tests (**d**, **e**, **k** and **l**) and the two-sided *χ*^2^ contingency statistic (**b**, **c** and **n**).

To examine the binding and neutralizing activity of the memory antibodies elicited by mRNA vaccination in C144-LS and C135-LS recipients, we produced 178 representative monoclonal antibodies obtained from five individuals as IgGs and tested them for binding to the WT SARS-CoV-2 RBD using ELISA (Fig. 3a–c and Supplementary Table 4). In contrast to the controls, from whom over 95% of the memory antibodies bound strongly to the RBD, monoclonal antibodies isolated from volunteers who received C144-LS and C135-LS showed diverse levels of binding activity. Approximately one-quarter (24%) of the antibodies displayed relatively poor binding, with ELISA half-maximal effective concentrations (EC_50_) that were only slightly above our limit of detection, and a little over one-third (38%) showed no detectable binding above the background (Fig. 3a,b). Accordingly, the median (EC_50_) of antibodies isolated from monoclonal antibody recipients was significantly higher than in the controls (Fig. 3b; *P* < 0.0001). Notably, this difference remained significant when the monoclonal antibodies isolated from IgM and IgG memory cells were analysed independently (Fig 3c; *P* = 0.0005 and *P* < 0.0001, respectively).

**Extended Data Fig. 5:**
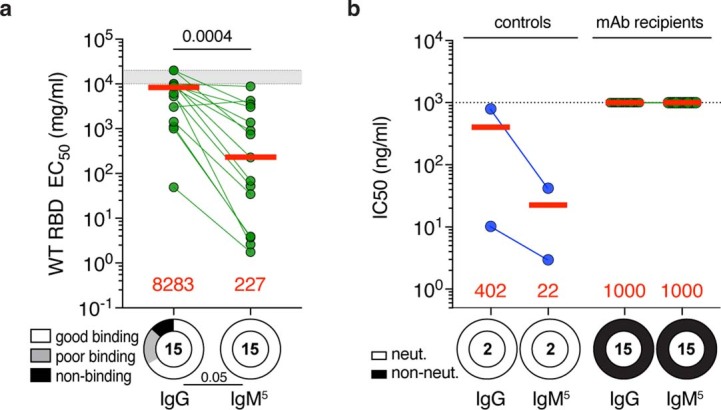
WT RBD binding and WT SARS-Cov-2 neutralization by monoclonal pentameric IgM antibodies. **a**-**b**, Panels depict WT RBD binding and WT SARS-CoV-2 S pseudotype neutralizing activity of a representative panels of monoclonal antibodies derived from IgM-expressing RBD-specific memory B cells expressed either as human IgG1 (IgG, as in Fig. 3a–c) or pentameric IgM (IgM^5^). **a**, Panel shows WT RBD EC50s of 15 monoclonal antibodies isolated from vaccinated mAb recipients (also see Supplementary Table 4). Grey shaded area between horizontal dotted lines indicates antibodies with EC50s >10 µg ml^−1^ (poor binding) and non-binding antibodies arbitrarily grouped at 10 and 20 µg ml^−1^, respectively. **b**, Plots show IC50s of 2 IgM-derived control antibodies (covering a wide range of neutralizing activity) in blue and 15 IgM-derived monoclonal antibodies from mAb recipients (as in **a**) in green, expressed as human IgG1 (IgG) or pentameric IgM (IgM^5^). For both panels (**a**, **b**), ring plots summarize the fraction of antibodies in the indicated category among all tested (encircled number). Red horizontal bars and numbers indicate median values. For panel **a**, statistical significance was determined using the two-tailed Wilcoxon matched-pairs rank test to compare differences between the same monoclonal antibodies expressed as IgG or pentameric IgM, and the Chi-squared contingency statistic was used to compare categorical distributions from ring plots.

Memory antibodies obtained from C144-LS and C135-LS recipients that bound to WT SARS-CoV-2 RBD with EC_50_ values of <10 µg ml^−1^ were tested for neutralizing activity against viruses pseudotyped with WT S. Whereas almost two-thirds (63%) of the IgG and 17% of the IgM antibodies isolated from controls showed measurable neutralizing activity, only 1 out of 45 IgG-derived antibodies and none of the 32 IgM-derived antibodies obtained from C144-LS and C135-LS recipients neutralized SARS-CoV-2 (Fig. 3d,e). Thus, the antibodies isolated from the RBD-specific memory B cell compartment of vaccinated monoclonal antibody recipients show significantly less neutralizing activity compared with the antibodies isolated from the controls. In circulation, IgM antibodies are pentamers that, in addition to their superior ability to fix complement, show increased apparent affinities. To test whether the binding and neutralizing properties of IgM antibodies would be improved when expressed as pentamers, we re-expressed 17 IgM memory antibodies (15 from monoclonal antibody recipients and 2 from controls) as pentameric IgMs. Although the pentamers obtained from monoclonal antibody recipients showed significantly improved binding to RBD by ELISA (Extended Data Fig. 5a and Supplementary Table 4; *P* = 0.0004), they remained unable to neutralize (Extended Data Fig. 5b and Supplementary Table 4). By contrast, both control IgM antibodies showed improved neutralizing potency when expressed as pentamers (Extended Data Fig. 5b).

To examine the affinity of the antibodies, we performed biolayer interferometry experiments (BLI) in which monoclonal antibodies were immobilized onto the biosensor chip and exposed to WT RBD monomers^12^ (Fig. 3f,g). In contrast to antibodies derived from the control individuals, for which 96% of the antibodies tested displayed measurable affinities, only two-thirds (67%) of the antibodies derived from monoclonal antibody recipients did so (Fig. 3g,i,k; *P* < 0.0001). When all of the antibodies were considered together, the median affinity (affinity constant (*K*_d_)) differed by nearly one order of magnitude between monoclonal antibody recipients and the controls (Fig. 3k; *P* < 0.0001). Moreover, this difference remained significant when IgM and IgG monoclonal antibodies were considered independently (Fig 3l; *P* = 0.0058 and *P* < 0.0001, respectively), indicating that the lower affinities observed in the memory compartment of monoclonal antibody recipients cannot solely be explained by the preponderance of IgM.

C144-LS and C135-LS have the potential to form immune complexes with the vaccine antigen in vivo and present it as a multimer that could increase the apparent affinity of a B cell for the multimerized antigen by avidity effects. To determine whether memory-cell-derived antibodies from monoclonal antibody recipients with no apparent affinity to monomeric antigen would show binding under higher valency conditions, we exposed the immobilized monoclonal antibodies to biotin–streptavidin tetramerized trimers of S (Fig. 3f,h,j). Of the 25 antibodies with no apparent monomeric binding tested, 23 (92%) bound to multimerized S (Fig. 3h,j). We conclude that most of the anti-RBD antibodies isolated from monoclonal antibody recipients that did not show detectable binding to RBD monomers bind to multimerized antigen. Thus, the absence of binding to monomeric antigen is a consequence of the relatively low affinity of the memory antibodies derived from monoclonal antibody recipients and not due to altered specificity.

**Extended Data Fig. 6:**
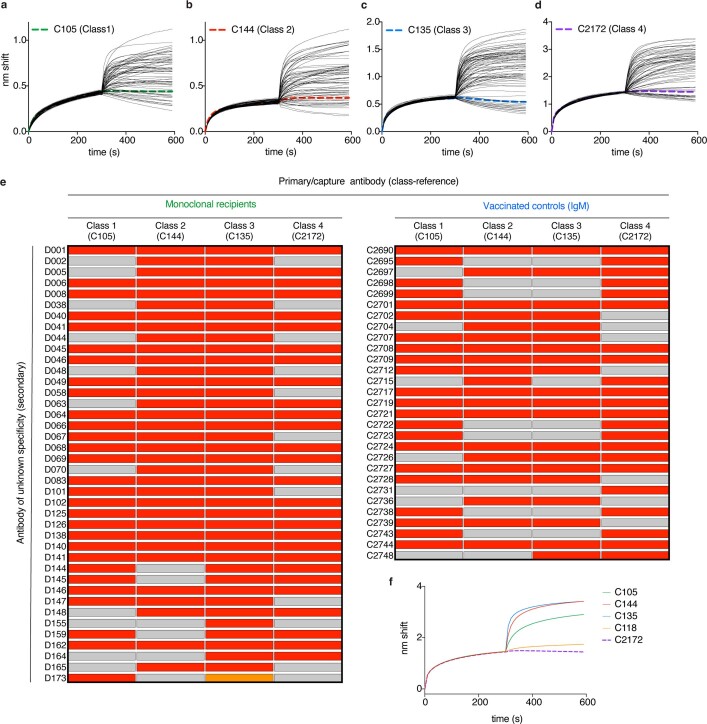
Competition BLI. **a**-**d**, BLI traces of antibodies assayed for competition with class-reference antibodies. Traces show initial association curve (antigen capture phase of the primary antibody) and subsequent addition of secondary antibodies of unknown class. Thin solid black lines represent antibodies isolated from mAb recipients or vaccinated controls. Thick dashed lines are self-competition traces of C105 (green in **a**), C144 (red in **b**), C135 (blue in **c**) and C2172 (purple in **d**) for classes 1–4, respectively. **e**, Heat-map of relative inhibition of secondary antibody binding to the preformed capture antibody-RBD complexes (grey=no binding, red=unimpaired binding, orange=indeterminate). The left panel shows antibodies from mAb recipients, while the right panel shows IgM antibodies from vaccinated controls isolated in this study (both after vax2). Details on IgG antibodies isolated from vaccinated controls can be found in^9,11^. **f**, BLI traces defining C2172 as Class 4. C2172 is the primary/capture antibody (in dashed purple). The addition of known class-defining antibodies C105 (in green, Class 1^8^), C144 (in red, Class 2^8^), C135 (in blue, Class 3^8^), and C118 (in orange, Class 1/4^27^) establish C2172 as a bona fide Class 4 antibody.

To examine the epitopes targeted by the vaccine-elicited anti-RBD antibodies produced by memory B cells of C144-LS and C135-LS recipients, we performed BLI experiments in which a preformed antibody–RBD complex comprising one of four structurally characterized antibodies^8,26,27^ was exposed to a second monoclonal antibody targeting an unknown epitope (Fig. 3m and Extended Data Fig. 6). A total of 49% of the anti-RBD memory antibodies obtained from vaccinated controls target class 1, 2 or 3 epitopes or combinations thereof^9,11^ (Fig. 3n and Extended Data Fig. 6a–f). By contrast, only 20% of the memory antibodies obtained from monoclonal antibody recipients targeted class 1 or 2 epitopes, and none were class 3 specific. Instead, we found that 78% of these antibodies targeted either class-4-containing epitopes or epitopes that could not be classified using our method (Fig. 3n and Extended Data Fig. 6a–f). Thus, there was a significant shift in the distribution of epitopes targeted by memory antibodies isolated from monoclonal antibody recipients compared with the controls (Fig. 3n; *P* = 0.0089). In conclusion, C144-LS and C135-LS alter the development of memory B cells expressing anti-RBD antibodies and their epitope target preference.

## Germinal centre B cell responses

**Fig. 4:**
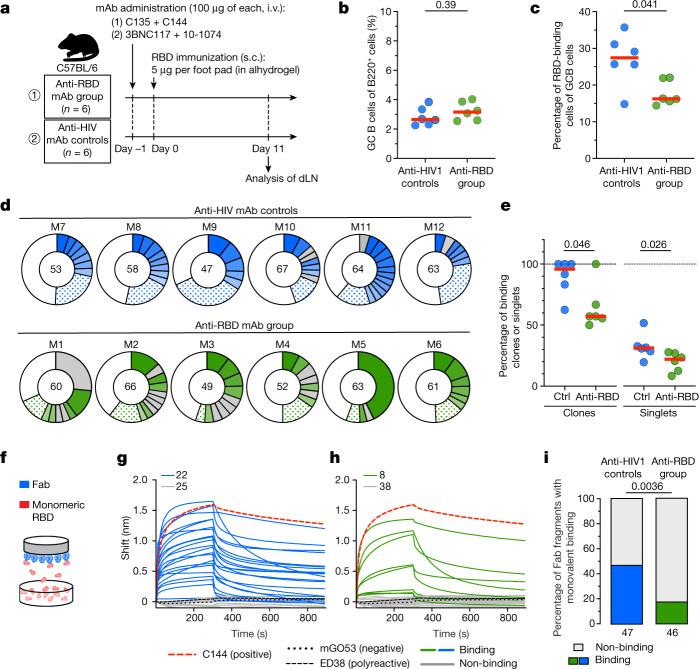
Germinal centre responses in mice pretreated with monoclonal antibodies. **a**, Schematic of the experimental set-up. Pooled popliteal lymph nodes (dLN) were analysed using flow cytometry (Extended Data Fig. 7 and Methods). **b**,**c**, Enumeration of germinal centre B cells (CD38^−^FAS^+^GL7^+^) as a fraction of all B220^+^ B cells (**b**) and RBD-binding cells as a fraction of germinal centre B (GCB) cells (**c**). **d**,**e**, Antibody sequences from single germinal centre B cells. **d**, The distribution of antibody sequences. The encircled numbers indicate the number of sequences analysed per animal. Solid pie chart slices indicate clonally expanded sequences, with slices coloured in shades of blue (controls) or green (anti-RBD monoclonal antibody group) indicating binding clones (Extended Data Fig. 7b and Supplementary Table 5). The grey slices denote non-binding clones. Sequences appearing only once are stippled (binding) or white (non-binding). **e**, The relative contribution of binding clones and singlets in **d**. **f**–**i**, Binding of monoclonal germinal centre B-cell-derived Fab fragments to monomeric RBD as determined using BLI. **f**, The BLI set-up. **g**,**h**, Traces of Fab fragments derived from controls (**g**) and anti-RBD-treated mice (**h**). Each curve represents one Fab. The coloured solid lines denote binding above the background represented by polyreactive antibody ED38^35^ (dashed black line) and negative control antibody mGO53 (dotted black line). The grey lines show non-binding antibodies. C144 (thick, red-dashed line) was used as the positive control. **i**, The percentage of binding Fab fragments, with the total number of Fab fragments tested from the control (*n* = 47) and anti-RBD monoclonal antibody group (*n* = 46) denoted below. For **b**–**i**, control mice pretreated with irrelevant anti-HIV monoclonal antibodies (3BNC117 and 10-1074, *n* = 6) are shown in blue, and mice that received a combination of C135 and C144 (*n* = 6) are shown in green. For **b**, **c** and **e**, the coloured dots represent individual mice and the red horizontal lines indicate the median values. Statistical significance was determined using two-tailed Mann–Whitney *U*-tests (**b**, **c** and **e**) and two-sided Fisher’s exact tests (**i**).

**Extended Data Fig. 7:**
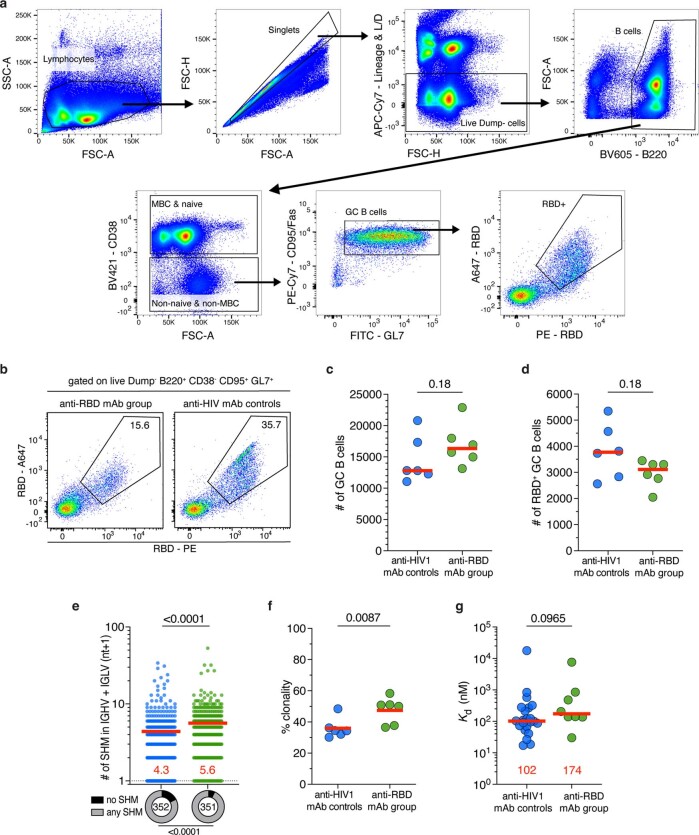
Flow-cytometry of germinal center responses in mAb pre-treated mice and molecular characterization of germinal-centre-derived monoclonal antibodies. **a**, Gating strategy for flow-cytometry phenotyping of germinal centre B cells isolated from the draining (popliteal) lymph nodes of WT C57BL/6 mice immunized with recombinant SARS-Cov-2 RBD 11 days prior (as illustrated in Fig. 4a). Gating was on single lymphocytes that were B220^+^ and CD4^−^ CD8a^−^ NK1.1^−^ F4/80^−^ (lineage-negative) without uptake of live-dead dye (L/D). Germinal centre B cells were those that were CD38^−^ GL7^+^ CD95 (Fas)^+^. Antigen-specific cells were those with dual binding to Wuhan-Hu1 RBD-PE and RBD-AF647. **b**, Representative flow-cytometry plots of Wuhan Hu-1 RBD-binding germinal centre B cells from mice that had either received the combination of C135 and C144 (anti-RBD mAb group) or the irrelevant anti-HIV control antibodies 3BNC117 and 10–1074 (anti-HIV mAb controls) one day prior to immunization, with the percentage of binding cells denoted within the respective gate. **c**-**d**, Panels show the total number of CD38^−^ GL7^+^ CD95 (Fas)^+^ germinal centre B cells (**c**) and RBD-binding germinal centre B cells (**d**) isolated from anti-RBD mAb pre-treated (n = 6, green) and control mice (n = 6, blue), with each dot corresponding to an individual mouse. **e**, Somatic hypermutation (SHM) levels of germinal centre B-cell-derived monoclonal antibodies shown as combined heavy- and light-chain variable region nucleotide substitutions plus one (IGVH+IGVL+1), with each dot representing one sequence from anti-RBD mAb pre-treated mice (green) or controls (blue). Ring plots below each column show the fraction of sequences with no (IGVH+IGVL+1 = 1) vs. any (IGVH+IGVL+1 > 1) SHM among all sequences analysed (encircled number) for the respective group. **f**, Percentage of sequences belonging to clones, defined as 2 or more sequences with the same IGHV and IGLV genes and with highly similar CDR3s, among all sequences obtained from the respective animal (as in Fig. 4d). Each dot represents one individual mouse from the anti-RBD mAb (n = 6, green) or control group (n = 6, blue). **g**, Affinity constants (*K*_d_) of germinal centre B-cell-derived Fabs for WT SARS-CoV-2 RBD, as established from the monovalent interaction of Fabs with RBD monomers by BLI (also see Fig. 4f–i, Supplementary Table 6 and methods). Each dot represents a single Fab from the anti-RBD mAb (n = 8, green) or control group (n = 22, blue). Red horizontal bars (**c**-**g**) and numbers (**e**, **g**) indicate median (**c**, **d**, **f**, **g**) and mean (**e**) values. Statistical significance was determined using the two-tailed Mann-Whitney test for **c**-**g d**, and the two-sided Fisher’s exact test was used to test the relative contribution of mutated and unmutated sequences in **e**.

To examine the mechanism by which pre-existing antibodies alter memory B cell selection, we immunized WT C57BL/6 mice that were preinfused with C135 and C144 or an irrelevant anti-HIV monoclonal antibody cocktail with recombinant SARS-CoV-2 RBD (Fig. 4a). Mice that were pretreated with control anti-HIV antibodies developed germinal centre responses in which an average of 27% of the B cells bound to RBD (Fig. 4b,c and Extended Data Fig. 7a–d). Although the germinal centre size was not altered in mice that received C135 and C144 (Fig. 4b and Extended Data Fig. 7c), the fraction of RBD-binding cells was significantly reduced (Fig. 4c and Extended Data Fig. 7b,d; *P* = 0.041).

To examine the molecular nature of the memory antibodies produced in the presence of pre-existing high-affinity antibodies, we cloned and produced antibodies from germinal centre B cells. A total of 351 and 352 antibodies were obtained from the anti-RBD-treated and anti-HIV-treated control groups, respectively (Supplementary Table 5). B cells isolated from both groups showed similar levels of somatic mutation (Extended Data Fig. 7e) and clonal expansion (Fig. 4d and Extended Data Fig. 7f). However, in the C135 and C144 recipients, clonally expanded and unique B cells were dominated by cells that did not bind to the RBD, as determined using flow cytometry (Fig. 4d,e; *P* = 0.046 and *P* = 0.026, respectively).

A total of 106 monoclonal antibodies were expressed as fragment antigen-binding (Fab) fragments (Supplementary Table 6) and tested for binding to SARS-CoV-2 RBD by BLI (Fig. 4f). Under monomeric binding conditions, 46% (22 out of 47) of the Fab fragments derived from the control mice bound to the RBD (Fig. 4g). By contrast, only 21% (8 out of 46) of the germinal centre B cell antibodies from the mice that were pretreated with C135 and C144 showed measurable but demonstrably lower affinity under these conditions (Fig. 4g–i; *P* = 0.0036 and Extended Data Fig. 7g). Thus, our mouse immunization experiments show that pre-existing high-affinity antibodies lower the affinity threshold for B cell participation in immune responses, and thereby provide a mechanistic explanation for the observation that the memory compartment in humans infused with C135 and C144 is dominated by low-affinity B cells.

## Discussion

Our experiments show that pre-existing antibodies alter the development of memory B cells in response to SARS-CoV-2 mRNA vaccination in humans. Consistent with a recent report, C144-LS and C-135-LS did not significantly interfere with the development of circulating antibodies that bind to epitopes outside of the target sites of the two monoclonal antibodies^13^. Moreover, although we found that endogenous neutralizing responses were reduced in monoclonal antibody recipients after one dose, this difference was no longer significant after two doses of mRNA vaccination. By contrast, anti-RBD-specific memory B cell development was profoundly altered. Specifically, the affinity threshold for entry into the memory and germinal centre B cell compartment in humans and mice was lowered, and there was a change in the epitopes targeted in the presence of pre-existing antibodies.

Memory cells expressing IgG antibodies specific to class 1, 2 or 3 epitopes normally dominate the anti-RBD response after 2 doses of mRNA vaccination^4–8^. By contrast, we found that recipients of monoclonal antibodies developed increased numbers of IgM anti-RBD memory cells that express antibodies with altered epitope specificity consistent with epitope masking. This may also explain the shift in memory B cell specificity away from class 1 and 2 after the third dose of the SARS-CoV-2 mRNA vaccines that increases the breadth of the neutralizing response^9^.

Beginning with experiments on anti-diphtheria toxin antibodies in the early part of the twentieth century, extensive work in experimental animals showed that passive transfer of polyclonal immune serum or monoclonal antibodies can alter subsequent humoral immune responses in an epitope-specific manner^1,2^. More recently, epitope masking by pre-existing antibodies was shown to interfere with the epitope-specific plasmablast response to malaria vaccination^28,29^. In malaria vaccine trials, a third vaccine dose produced a smaller fraction of high-affinity antibody-producing plasmablasts than the second, and this was attributed to epitope masking. The masking effect of pre-existing high-affinity antibodies was confirmed in transgenic mice, showing that high-affinity antibodies block B cell entry into germinal centres in an epitope-specific manner^29–31^. However, these results were obtained in the context of a restricted transgenic B cell repertoire and the effect of pre-existing antibodies on the development of memory B cells in polyclonal responses was not examined^29^. The results of our clinical trial and mouse experiments agree with the observation that pre-existing antibodies block the development of epitope-specific B cells^32,33^. Our experiments extend previous observations because we also examined the specificity and antigen-binding affinity of the antibodies produced by B cells that do respond to antigen in the presence of pre-existing antibody. Notably, we found that pre-existing high-affinity antibodies lower the affinity threshold for B cell participation in the immune response. As a result, the anti-SARS-CoV2 antibodies expressed by memory B cells developing in humans who received monoclonal antibody infusions before vaccination are dominantly of low affinity. By contrast, low-affinity polyclonal antibodies emerging after a vaccine prime may enhance vaccine booster responses by mechanisms that have not yet been fully elucidated^2,3,31^.

Memory B cells can develop through two different pathways^18,19^. Class-switched memory B cells that carry relatively higher-affinity antibodies with high numbers of somatic mutations develop in germinal centres. By contrast, IgM-expressing memory B cells that carry lower-affinity antibodies and only small numbers of mutations develop through a germinal-centre-independent pathway^18,20^. Our human data suggest that the passive transfer of C144-LS and C135-LS may favour the germinal-centre-independent pathway by creating immune complexes (reviewed in ref. ^34^). Immune complexes trap and sequester antigen in lymphoid organs and thereby increase local antigen concentration. Furthermore, they contain multiple copies of the antigen in a form that increases apparent affinity by avidity effects, thereby enabling the recruitment of B cells with very low-affinity receptors into the immune response^34^. Together, the increased antigen concentration and avidity effects combine to reduce selection stringency and help to explain the increase in low-affinity RBD-binding B cells found in human monoclonal antibody recipients, as this allows for the activation of greater numbers of abundant lower-affinity clones that would otherwise not be recruited to the immune response. Thus, the combination of epitope masking and lowered affinity thresholds diversify the ensuing B cell responses.

In conclusion, the composition of germinal centres and the development of memory in response to vaccination are influenced by pre-existing antibodies that can alter the antibody target profile, affinity, and isotype of the responding cells. Diversification of the antibody response through this mechanism may help to increase the breadth of vaccines like the SARS-CoV-2 vaccine but interfere with the development of breadth and potency in others, like HIV-1 or influenza, by diverting immunity away from broadly neutralizing to strain-specific epitopes and by allowing increasing numbers of low-affinity precursors expressing off-target antibodies to participate in the immune response.

## Methods

### Study participants

The participants in the monoclonal antibody recipient group were healthy SARS-CoV-2-naive volunteers who were enrolled in a first-in-human phase 1 study at the Rockefeller University Hospital in New York and received single doses of two anti-SARS-CoV-2 RBD monoclonal antibodies, C144-LS and C135-LS, between 13 January and 3 March 2021 (NCT04700163). The phase 1 clinical trial had a dose-escalation design and evaluated the safety and tolerability as well as the pharmacokinetics of the two monoclonal antibodies. The monoclonal antibody cocktail (1:1 ratio of C144-LS and C135-LS) was administered to 21 out of the 23 enrolled individuals (*n* = 2 receiving placebo), allowing for multiple interim safety analyses. The monoclonal antibodies were administered at 100 or 200 mg each s.c., or at 1, 5 or 15 mg per kg each i.v. (Supplementary Table 2). Eligible participants for the phase 1 study were healthy adults with no history of SARS-CoV-2 infection or vaccination, or previous receipt of any SARS-CoV-2 therapeutics, including other monoclonal antibodies or plasma from convalescent individuals. Further details on the inclusion and exclusion criteria, study design and end points of the phase 1 study can be found at ClinicalTrials.gov (NCT04700163). Of the 21 individuals who received the monoclonal antibodies in the phase 1 study, 18 co-enrolled in a parallel observational study to assess their immune responses to subsequent SARS-CoV-2 mRNA vaccination. One individual chose to receive the Janssen (Ad26.COV2.S) vaccine, and another individual (a placebo recipient) displayed N titre changes before enrolment in the observational study that were compatible with a recent SARS-CoV-2 infection, making them ineligible for inclusion in this study. The remaining phase 1 study participants chose not to enrol in the parallel study of immune responses. The 18 monoclonal antibody recipients included in this observational study received either the Moderna (Spikevax, mRNA-1273) or Pfizer-BioNTech (Comirnaty, BNT162b2) mRNA vaccines against the WT (Wuhan-Hu-1) strain of the severe acute respiratory syndrome coronavirus 2 (SARS-CoV-2). The participants in the vaccinated control group were healthy SARS-CoV-2-naive volunteers who had received two doses of one of the two currently approved SARS-CoV-2 mRNA vaccines, Moderna (Spikevax, mRNA-1273) or Pfizer-BioNTech (Comirnaty, BNT162b2). These control individuals had been recruited to the Rockefeller University Hospital for serial blood donations to longitudinally assess their immune responses to SARS-CoV-2 mRNA vaccination^9,11^. We previously reported the findings obtained from this group and refer to refs. ^9,11^ for further details on participant recruitment, inclusion and exclusion criteria, and demographic characteristics (Supplementary Tables 1 and 2). At each sample collection visit, the participants of either group presented to the Rockefeller University Hospital for blood sample collection and were asked to provide details of their vaccination regimen, possible side effects, comorbidities and possible COVID-19 history. Vaccinations were administered outside of the study, at the discretion of the individual and their health care provider consistent with existing guidelines and, as such, were not influenced by their participation in our study. Baseline and longitudinal plasma samples were tested for binding activity to the nucleocapsid protein (N; Sino Biological, 40588-V08B) of SARS-CoV-2. The absence of seroconversion towards N during the study interval was used to exclude SARS-CoV-2 infection, in addition to participants’ reported history. Clinical data collection and management were carried out using the software iRIS by iMedRIS (v.11.02). All of the participants provided written informed consent before participation in the study, which was conducted in accordance with good clinical practice. The study was performed in compliance with all of the relevant ethical regulations, and the clinical protocols (CGA-1015 and DRO-1006) for studies with human participants were approved by the Institutional Review Board of the Rockefeller University. Detailed participant characteristics are provided in Supplementary Tables 1 and 2 and refs. ^9,11^.

### Blood sample processing and storage

Peripheral blood mononuclear cells obtained from samples collected at Rockefeller University were purified as previously reported by gradient centrifugation and stored in liquid nitrogen in the presence of fetal calf serum (FCS) and dimethylsulfoxide^5^. Heparinized plasma and serum samples were aliquoted and stored at −20 °C or less. Before the experiments, aliquots of plasma samples were heat-inactivated (at 56 °C for 1 h) and then stored at 4 °C.

### Pharmacokinetics of C144-LS and C135-LS

To evaluate the pharmacokinetic properties of the passively administered antibodies C144-LS and C135-LS, their serum antibody levels were measured on the day of antibody administration (day 0) at 1, 3, 6, 9 and 12 h after infusion, and on days 1, 3, 7, 14, 21, 28, 56, 84, 126, 168, 252 and 336. C144-LS and C135-LS levels in the serum were measured using tandem mass spectrometry (MS/MS). In brief, analytes were isolated from serum samples through immunocapture using streptavidin beads and biotinylated RBD protein. The isolated proteins were denatured with dithiothreitol, alkylated with iodoacetamide, and digested with trypsin. The final extract was analysed using high-performance liquid chromatography with column-switching and MS/MS detection using positive-ion electrospray. A linear, 1/concentration^2^-weighted, least-squares regression algorithm was used for quantification. Non-compartmental analysis was used to estimate the pharmacokinetic parameters from measured serum levels of C144-LS and C135-LS. Phoenix WinNonlin (v.8.2) was used for the non-compartmental analysis. The actual sample time after the administration of each monoclonal antibody was used for the estimation of serum pharmacokinetic parameters instead of nominal time. Half-life estimates were similar between administration routes for both C144-LS and C135-LS, indicating a half-life of 2–3 months for both monoclonal antibodies by either administration route (C144-LS: 68.9 to 99.3 days for s.c. groups and 86.9 to 92.3 days for i.v. groups; C135-LS: 72.7 to 77.9 days for s.c. groups and 70.5 to 94.7 days for i.v. groups). Visualization of the pharmacokinetic data was performed in GraphPad Prism, using the three-phase decay model.

### Mouse immunization

C57BL/6J mice were purchased from Jackson. All of the mice used were females aged 6–12 weeks at the start of the experiments. The mice were housed at a temperature of 22 ºC and a humidity of 30–70% under a 12 h–12 h light–dark cycle with ad libitum access to food and water. Footpad immunizations were performed using 25 µl of 1× PBS containing 5 µg of recombinant RBD and 8.5 µl of 2% Alhydrogel (Invivogen, 21645-51-2). 3BNC117/10-1074 and C135/C144 antibody cocktails were prepared with 100 μg of each antibody and delivered in 1× PBS through i.v. injection. All of the animal procedures and experiments were performed without blinding or randomization, and according to protocols approved by the Rockefeller University Institutional Animal Care and Use Committee (IACUC).

### ELISAs

ELISA assays^36,37^ to evaluate antibody binding to SARS-CoV-2 Wuhan-Hu-1 RBD, NTD or S were performed by coating high-binding 96-half-well plates (Corning 3690) with 50 μl per well of a 1 μg ml^−1^ protein solution in phosphate-buffered saline (PBS) overnight at 4 °C. The plates were washed six times with washing buffer (1× PBS with 0.05% Tween-20 (Sigma-Aldrich)) and incubated with 170 μl per well blocking buffer (1× PBS with 2% BSA and 0.05% Tween-20 (Sigma-Aldrich)) for 1 h at room temperature. Immediately after blocking, monoclonal antibodies or plasma samples were added in PBS and incubated for 1 h at room temperature. Plasma samples were assayed at a 1:66 starting dilution and serially diluted by either threefold or fourfold. Monoclonal antibodies were tested at a starting concentration of 10 μg ml^−1^ and 11 additional threefold serial dilutions. The plates were washed six times with washing buffer and then incubated with anti-human IgG or IgM secondary antibody conjugated to horseradish peroxidase (HRP) (Jackson Immuno Research, 109-036-088 and 109-035-129) in blocking buffer at a 1:5,000 dilution (IgM and IgG). The plates were developed by addition of the HRP substrate, 3,3′,5,5′-tetramethylbenzidine (Thermo Fisher Scientific) for 6 min. The developing reaction was stopped by adding 50 μl of 1 M H_2_SO_4_ and absorbance was measured at 450 nm using an ELISA microplate reader (FluoStar Omega, BMG Labtech) with Omega and Omega MARS software for analysis. Normalizer control samples were included on each plate. For plasma samples and monoclonal antibodies, half-maximal binding titres (BT_50_) and half-maximal effective concentrations (EC_50_), respectively, were calculated using a four-parameter nonlinear regression model (GraphPad Prism v.9.3) with the following settings: [agonist] vs. response -- variable slope (four parameters); bottom = 0; Hillslope > 0; top = experiment-specific upper plateau of the normalizer control antibody or plasma sample reaching saturation for at least 3 consecutive dilution steps. The curve fit was constrained to an upper limit that corresponds to the maximal optical density achieved by the known normalizer control to limit interplate/interexperiment variability (batch effects). Pentameric IgM BT_50_ values were established using previously measured IgG antibodies as normalizer controls. Pre-pandemic plasma samples from healthy donors and isotype control monoclonal antibodies were used as negative controls as indicated and were used for validation^5^. All of the reported EC_50_ and BT_50_ values are the average of at least two independent experiments.

### Proteins

The mammalian expression vector encoding the RBD of SARS-CoV-2 (GenBank MN985325.1; S protein residues 319–539) was previously described^26^. Plasmids encoding the R346S/E484K and N440K/E484K substitutions were generated using a site-directed mutagenesis kit according to the manufacturer’s instructions (New England Biolabs (NEB), E0554S). All of the constructs were confirmed by Sanger sequencing and were used to express soluble proteins by transiently transfecting Expi293F cells (Gibco/Thermo Fisher Scientific, A14527)). Supernatants were collected after 4 days, and RBD proteins were purified by nickel affinity chromatography. S 6P proteins were purified by nickel affinity followed by size-exclusion chromatography. The peak fractions from size-exclusion chromatography were identified using native gel electrophoresis, and the peak fractions corresponding to monomeric RBDs or S trimers were pooled and stored at 4 °C.

### SARS-CoV-2 pseudotyped reporter virus

A plasmid expressing SARS-CoV-2 S in the context of pSARS-CoV-2-S_Δ19_ (Wuhan-Hu-1) has been described previously^6^, and a derivative of pSARS-CoV-2-S_Δ19_ with a disrupted furin-cleavage site was generated by introducing the R683G substitution^6^. Disruption of the furin-cleavage site results in increased particle infectivity. A plasmid expressing SARS-CoV-2 Omicron BA.4/5 S carrying the R683G substitution has been described previously^38^. Two plasmids containing C135/C144 antibody escape mutations were constructed on the basis of pSARS-CoV-2-S_Δ19_ R683G by overlap extension PCR-mediated mutagenesis and Gibson assembly. Specifically, the substitutions introduced were as follows: R346S/Q493K and R346S/N440K/E484K. Importantly, as those substitutions were incorporated into the pSARS-CoV-2-S_Δ19_ R683G background, neutralizing activity against all mutant and variant pseudoviruses were compared to a WT SARS-CoV-2 S sequence (GenBank: NC_045512) also carrying R683G (pSARS-CoV-2-S_Δ19_ R683G) where appropriate, as indicated. SARS-CoV-2 pseudotyped particles were generated as previously described^5,14^. In brief, HEK293T cells were transfected with pNL4-3ΔEnv-nanoluc and pSARS-CoV-2-S_Δ19_, and particles were collected 48 h after transfection, filtered and stored at −80 °C.

### Pseudotyped virus neutralization assay

Fivefold serially diluted pre-pandemic negative control plasma from healthy donors (technical negative controls, data not shown), plasma from vaccinated monoclonal antibody recipients and mRNA vaccinated controls, or monoclonal antibodies were incubated with SARS-CoV-2 pseudotyped virus for 1 h at 37 °C. The mixture was subsequently incubated with HEK293T-ACE2 cells^5^ (for all monoclonal antibody WT neutralization assays) or HT1080Ace2 cl14 cells^6^ (for all plasma neutralization assays) for 48 h, after which cells were washed with PBS and lysed with Luciferase Cell Culture Lysis 5× reagent (Promega). Nanoluc luciferase activity in the lysates was measured using the Nano-Glo Luciferase Assay System (Promega) with the ClarioStar Multimode reader (BMG, v.5.70.R3). The relative luminescence units were normalized to those derived from cells infected with SARS-CoV-2 pseudotyped virus in the absence of plasma or monoclonal antibodies. The half-maximal neutralization titre for plasma (NT_50_) or half-maximal and 90% inhibitory concentrations for monoclonal antibodies (IC_50_ and IC_90_) were determined using four-parameter nonlinear regression (least-squares regression method without weighting; constraints: top=1, bottom=0; in GraphPad Prism).

### Biotinylation of viral protein for use in flow cytometry and BLI

Purified and Avi-tagged SARS-CoV-2 Wuhan-Hu-1 RBD or S were biotinylated using the Biotin-Protein Ligase-BIRA kit according to manufacturer’s instructions (Avidity) as described previously^5^. Ovalbumin (Sigma-Aldrich, A5503-1G) was biotinylated using the EZ-Link Sulfo-NHS-LC-Biotinylation kit according to the manufacturer’s instructions (Thermo Fisher Scientific). Biotinylated ovalbumin was conjugated to streptavidin-BV711 for human single-cell sorts (BD Biosciences, 563262) or to streptavidin-BB515 for phenotyping (BD Biosciences, 564453). For all human and mouse experiments, RBD was conjugated to streptavidin-PE (BD Biosciences, 554061) and streptavidin-AF647 (BioLegend, 405237)^5,9^.

### Human flow cytometry and single-cell sorting

Single-cell sorting by flow cytometry was described previously^5^. In brief, peripheral blood mononuclear cells were enriched for B cells by negative selection using a pan-B-cell isolation kit according to the manufacturer’s instructions (Miltenyi Biotec, 130-101-638). Before staining, the enriched B cells were incubated with an FcR-blocking antibody (BD, 564220) at a 1:200 dilution in fluorescence-activated cell sorting (FACS) buffer (1× PBS, 2% FCS, 1 mM ethylenediaminetetraacetic acid (EDTA)) for 20 min on ice. Subsequently, cells were incubated in FACS buffer with the following anti-human antibodies (all at 1:200 dilution): anti-CD20-PECy7 (BD Biosciences, 335793), anti-CD3-APC-eFluro 780 (Invitrogen, 47-0037-41), anti-CD8-APC-eFluor 780 (Invitrogen, 47-0086-42), anti-CD16-APC-eFluor 780 (Invitrogen, 47-0168-41), anti-CD14-APC-eFluor 780 (Invitrogen, 47-0149-42) and Zombie NIR (BioLegend, 423105), and fluorophore-labelled RBD and ovalbumin (Ova) for 30 min on ice. AccuCheck Counting Beads (Life Technologies, PCB100) were added as indicated to each sample according to manufacturer’s instructions. Single CD3^−^CD8^−^CD14^−^CD16^−^CD20^+^Ova^−^RBD–PE^+^RBD−AF647^+^ B cells were sorted into individual wells of 96-well plates containing 4 μl of lysis buffer (0.5× PBS, 10 mM dithiothreitol, 3,000 U ml^−1^ RNasin Ribonuclease Inhibitors (Promega, N2615) per well using the FACS Aria III system and FACSDiva software (Becton Dickinson) for acquisition and FlowJo for analysis. The sorted cells were frozen on dry ice, and then stored at −80 °C for subsequent RNA reverse transcription. For B cell phenotype analysis, in addition to above antibodies, B cells were also stained with following anti-human antibodies (all at 1:200 dilution): anti-CD19-BV605 (BioLegend, 302244), anti-IgG-PECF594 (BD, 562538), anti-IgM-AF700 (BioLegend, 314538) and anti-CD38-BV421 (BioLegend, 303526).

### Mouse flow cytometry and single-cell sorting

Popliteal lymph nodes from mice were collected in FACS buffer (1× PBS, 2% FBS, 2 mM EDTA), mechanically disrupted and filtered through a 35 µM strainer (Corning, 352235). Cells then underwent iterative rounds of staining each for 20 min on ice (all of the antibodies were diluted at 1:200 unless stated otherwise): (1) anti-mouse CD16/32 (Mouse BD FC Block, BD Biosciences, 553142); (2) fluorophore-conjugated RBD (see above); (3) anti-T and anti-B cell activation antigen-FITC (BD Biosciences, 553666), anti-CD38-PB (BioLegend, 102720; 1:100 dilution), anti-CD45R/B220-BV605 (BioLegend, 103244), anti-CD4-APC-eFluor780 (Invitrogen, 47-0042-82), anti-CD8a-APC-eFluor780 (Invitrogen, 47-0081-82), anti-NK1.1-APC-eFluor780 (Invitrogen, 47-5941-82), anti-F4/80-APC-eFluor780 (Invitrogen, 47-4801-82), anti-CD95-PE-Cy7 (BD Biosciences, 557653) and Zombie NIR (BioLegend, 423105, 1:1,000). AccuCheck Counting Beads (Life Technologies, PCB100) were added to samples according to the manufacturer’s instructions. Single CD4^−^CD8a^−^NK1.1^−^F4/80^−^B220^+^CD38^−^GL7^+^CD95^+^ cells were sorted on the BD FACSymphony S6 system into 96-well plates containing 1% 2-β-mercaptoethanol (Sigma-Aldrich) in TCL Buffer (Qiagen, 1031576) and subsequently frozen on dry ice and stored at −80 °C for RNA reverse transcription.

### Antibody sequencing, cloning and expression

Human antibodies were identified and sequenced as described previously^5,39^. In brief, RNA from single cells was reverse-transcribed (SuperScript III Reverse Transcriptase, Invitrogen, 18080-044) and the cDNA was stored at −20 °C or used for subsequent amplification of the variable *IGH*, *IGL* and *IGK* genes by nested PCR and Sanger sequencing. Sequence analysis was performed using MacVector (v.17.5.4) and Geneious Prime (v.2020.1.2 and v.2022.1.1). Amplicons from the first PCR reaction were used as templates for sequence- and ligation-independent cloning into antibody expression vectors. Mouse monoclonal antibodies were sequenced and cloned from single FACS-sorted B cells as previously described^40,41^ with the following modifications. In brief, RNA from single cells in 96-well plates was purified using magnetic beads (RNAClean XP, Beckman Coulter, A63987). Single-cell RNA was eluted from magnetic beads with 11 μl of solution containing 14.4 ng μl^−1^ of random primers (Invitrogen, 48190011), 0.5% of IGEPAL CA-630 (10% in distilled H–O, Sigma-Aldrich, I8896-50ML) and 0.6 U μl^−1^ of RNase inhibitor (Promega, N2615) in nuclease-free water (Qiagen, 129115), followed by incubation at 65 °C for 3 min. cDNA was synthesized by reverse transcription (SuperScript III Reverse Transcriptase 10,000 U, Invitrogen, 18080-044) and stored at −20 °C after addition of 10 μl nuclease-free water. Subsequent amplification of the variable *IGH* and *IGK* antibody genes was achieved by nested PCR using HotstarTaq DNA polymerase (Qiagen, 203209), using previously published primers^41^ and the following thermocycler conditions for annealing (see details below), elongation (all 72  ºC/55 s) and number of cycles: PCR1 (*IGG*, *IGM* and *IGK*): 46 °C/30 s/50 cycles; PCR2 (*IGG* and *IGM*): 55 °C/30 s/50 cycles; PCR2 (*IGK*): 46 °C/30 s/50 cycles. After purification, heavy chain and light chain PCR products were Sanger sequenced, and subsequently analysed using MacVector and Geneious Prime (v.2022.1.1), as well as the bioinformatics pipeline detailed below. Mouse Ig sequences were ordered as eBlocks (IDT) with short homologies for sequence and ligation independent cloning and cloned into human IGHG1 Fab and IGLK expression vectors as previously described^41^. All plasmid sequences were verified by Sanger sequencing, and all recombinant monoclonal antibodies (human memory B cell derived full-length IgG and His_6_-tagged mouse-derived human IgG1 Fab fragments) were thereafter produced and purified as previously described^5,41^. To produce pentameric IgMs, cloning from PCR products was performed by sequencing and ligation-independent cloning as described above, except that the *IGH* variable gene was cloned into an Igμ expression vector (InvivoGen, pfusess-hchm3). Pentameric IgMs were then expressed through transient transfection of HEK293-6E cells with vectors encoding the appropriate J chain, light chain and heavy chain at a ratio of 1:1.5:1.5. Secreted IgMs were collected from cell supernatants after 6 days and purified using the POROS CaptureSelect IgM Affinity Matrix kit (Thermo Fisher Scientific, 1952890500). Affinity-purified IgMs were further purified by size-exclusion chromatography. Peak fractions of pentameric IgMs were analysed using SDS–PAGE, pooled and buffer-exchanged into PBS using an Amicon Ultra 100 kDa (Millipore) centrifugal filter unit.

### BLI analysis

BLI assays were performed as previously described^5,15,41^ with minor modifications as below. In brief, we used the ForteBio Octet Red instrument (ForteBio Data Acquisition software v.11.1.3.25) at 30 °C with shaking at 1,000 r.p.m. Monomeric affinities of anti-SARS-CoV-2 RBD IgG and Fab fragments to RBD were derived by subtracting the signal obtained from traces performed with the same IgG/Fab in the absence of WT RBD. Kinetic analysis using protein A biosensor (ForteBio, 18-5010) for human IgGs was performed as follows: (1) baseline: immersion for 60 s in buffer (1× Octet Kinetic buffer, Sartorius, 18-1105); (2) loading: immersion for 200 s in a solution with IgGs at 10 μg ml^−1^; (3) baseline: immersion for 200 s in buffer; (4) association: immersion for 300 s in solution with WT RBD at three different concentrations ranging from 200 to 5 μg ml^−1^; (5) dissociation: immersion for 600 s in buffer. Curve fitting was performed using a fast 1:1 binding model and the data analysis software from ForteBio. Mean equilibrium dissociation constants (*K*_d_) were determined by averaging all binding curves that matched the theoretical fit with an *R*^2^ value of ≥0.8. To establish binding of low-affinity antibodies to multimerized antigen, 6P-stabilized and biotinylated S trimers were incubated with recombinant streptavidin (ACROBiosystems, STN-N5116) for 30 min at room temperature, resulting in up to 12 RBD-binding moieties per molecule and assayed on the Octet Red instrument (ForteBio) as described above, with the following modification: association step (4) was performed with the S-multimer at 430 μg ml^−1^. Epitope mapping assays were performed with protein A biosensor (ForteBio, 18-5010), according to the manufacturer’s protocol ‘classical sandwich assay’ as follows: (1) sensor check: sensors were immersed for 30 s in buffer alone (buffer ForteBio, 18-1105); (2) capture first antibody: sensors were immersed for 10 min with antibody 1 at 10 μg ml^−1^; (3) baseline: sensors were immersed 30 s in buffer alone; (4) blocking: sensors were immersed for 5 min with IgG isotype control (3BNC117) at 20 μg ml^−1^; (5) baseline: sensors were immersed for 30 s in buffer alone; (6) antigen association: sensors were immersed for 5 min with RBD at 20 μg ml^−1^; (7) baseline: sensors were immersed for 30 s in buffer alone. (8) Association antibody b2: sensors were immersed for 5 min with antibody 2 at 10 μg ml^−1^. Affinity testing of mouse germinal centre B-cell-derived human IgG1 Fab fragments was performed using the same steps and Octet Red instrument (ForteBio) settings as for the human memory-derived full-length IgG antibodies (see above) with the following modifications: monoclonal Fab fragments at 50 μg ml^−1^ were captured on FAB2G biosensors (Sartorius 18-5125) for step 2; monovalent RBD was added at concentrations ranging from 30 to 120 μg ml^−1^ in step 4. Binding Fab fragments with measurable affinities were defined as having biologically plausible association and dissociation kinetics (that is, being able to associate to saturation on the biosensor in step 2 and showing a discernible association and dissociation of antigen after steps (4) and (5)) as well as a computed *K*_d_ value that matched the theoretical fit with an *R*^2^ value of ≥0.75. Affinities of Fab fragments that did not get captured on the biosensor to saturation at the concentration tested could not be resolved and were excluded from further analysis (marked as N/D in Supplementary Table 6). In all cases, curve fitting was performed using the ForteBio Octet Data analysis software (ForteBio Data Analysis HT v.11.1.3.50).

### Computational analyses of antibody sequences

Antibody sequences were trimmed based on quality and annotated using Igblastn v.1.14 with the IMGT domain delineation system. Annotation was performed systematically using Change-O toolkit (v.0.4.540)^42^. The clonality of the heavy and light chain was determined using DefineClones.py implemented by Change-O (v.0.4.5)^42^. The script calculates the Hamming distance between each sequence in the dataset and its nearest neighbour. Distances are subsequently normalized to account for differences in junction sequence length, and clonality is determined based on a cut-off threshold of 0.15. Heavy and light chains derived from the same cell were subsequently paired, and clonotypes were assigned on the basis of their *V* and *J* genes using custom R and Perl scripts. All scripts and the data used to process antibody sequences are publicly available at GitHub (https://github.com/stratust/igpipeline/tree/igpipeline2_timepoint_v2). The frequency distribution of human *V* genes in anti-SARS-CoV-2 antibodies from this study (Extended Data Fig. 4f–h) was compared to 131,284,220 IgH and IgL sequences generated in ref. ^43^ and downloaded from cAb-Rep^44^—a database of human shared BCR clonotypes available online (https://cab-rep.c2b2.columbia.edu/). On the basis of the 108 distinct *V* genes that make up the 417 analysed sequences from the Ig repertoire of the individuals described in this study (353 sequences isolated form 5 monoclonal antibody recipients and 65 IgM sequences isolated from 9 vaccinated control individuals (for IgG sequences isolated from controls, see refs. ^9,11^), we selected the IgH and IgL sequences from the database that are partially coded by the same *V* genes and counted them according to the constant region. The frequencies shown in Extended Data Fig. 4f–h are relative to the source and isotype analysed. We used two-sided binomial tests to check whether the number of sequences belonging to a specific *IGHV* or *IGLV* gene in the repertoire was different according to the frequency of the same *IGV* gene in the database. Adjusted *P* values were calculated using correction for false-discovery rate. Significant differences are denoted by asterisks. Nucleotide somatic mutations and complementarity-determining region (CDR3) length were determined using custom R and Perl scripts. For quantification of somatic mutations, the *IGHV* and *IGLV* nucleotide sequences were aligned against their closest germlines using Igblastn and the number of differences was considered to be the number of nucleotide mutations.

### Data presentation

Figures were arranged in Adobe Illustrator 2022.

### Reporting summary

Further information on research design is available in the Nature Portfolio Reporting Summary linked to this article.

## Online content

Any methods, additional references, Nature Portfolio reporting summaries, source data, extended data, supplementary information, acknowledgements, peer review information; details of author contributions and competing interests; and statements of data and code availability are available at 10.1038/s41586-022-05609-w.

## Supplementary information

Supplementary Information Descriptions for Supplementary Tables 1–6.

Reporting Summary

Supplementary Table 1 Cohort characteristics.

Supplementary Table 2 Individual participant characteristics.

Supplementary Table 3 Sequences of human anti-SARS-CoV-2 RBD antibodies.

Supplementary Table 4 Sequences, RBD ELISA binding, neutralization, affinity and epitopes of cloned recombinant human anti-SARS-CoV-2 RBD antibodies.

Supplementary Table 5 Sequences of mouse germinal centre B cell antibodies.

Supplementary Table 6 Sequences and binding affinities of cloned recombinant monoclonal mouse germinal centre Fab fragments.

## Data Availability

Data are provided in Supplementary Tables 1–6. The raw sequencing data and computer scripts associated with Figs. 2 and 4 and Extended Data Figs. 3 and 7 have been deposited at GitHub (https://github.com/stratust/igpipeline/tree/igpipeline2_timepoint_v2). This study also uses data from ‘A public database of memory and naive B-cell receptor sequences’ (10.5061/dryad.35ks2), the Protein Data Bank (6VYB and 6NB6), cAb-Rep (https://cab-rep.c2b2.columbia.edu/), the Sequence Read Archive (SRP010970) and from ‘High frequency of shared clonotypes in human B cell receptor repertoires’ (10.1038/s41586-019-0934-8).

## Code availability

Computer code to process the antibody sequences is available at GitHub (https://github.com/stratust/igpipeline/tree/igpipeline2_timepoint_v2).
