# Supplementary material for: Antibody feedback regulates immune memory after SARS-CoV-2 mRNA vaccination
Source: Nature. 2022 Dec 6;613(7945):735–42. doi: 10.1038/s41586-022-05609-w (PMC9876794; doi:10.1038/s41586-022-05609-w)
Supplement: Supplementary file 1 — Descriptions for Supplementary Tables 1–6. [file 41586_2022_5609_MOESM1_ESM.pdf]

nature portfolio

https://doi.org/10.1038/s41586-022-05609-w

Supplementary information

Antibody feedback regulates immune memory after SARS-CoV-2 mRNA vaccination

In the format provided by the authors and unedited

Supplementary Guide

Supplementary Table 1: Cohort characteristics.

This table lists the characteristics of the monoclonal antibody (mAb) recipient group (top) and the control group consisting of individuals who were vaccinated but who had not received monoclonal antibodies prior (bottom). Reported data are median (range) unless stated otherwise.

Supplementary Table 2: Individual participant characteristics.

This table lists various characteristics (demographic, related to mAb and vaccine administration, and serological parameters) for individual study participants. Individuals from whom single RBD-binding memory B cells were isolated, sequenced, and representative antibodies cloned and tested are denoted with asterisks (*). Male and female sex are denoted as M and F, respectively. Control cohort individuals were previously published by Wang et al. (https://doi.org/10.1038/s41586-021-03324-6), and/or Cho et al. (https://doi.org/10.1038/s41586-021-04060-7), and/or Muecksch et al. (https://doi.org/10.1038/s41586-022-04778-y). For mAb recipients, the route of administration was either by subcutaneous injection (s.c.) or via intravenous infusion (i.v.).

Supplementary Table 3: Sequences of human anti-SARS-CoV-2 RBD antibodies.

This table summarizes the antibody sequences of single FACS-sorted RBD-binding B cells isolated after 2 doses of mRNA vaccination (vax2) as shown in Fig. 2d-g and Ext. Data Fig. 4. Sequences derived from all individuals of the respective group are shown in the respective sheets, as indicated. Clonally related sequences (same V and J genes at Heavy and Light Chains and similar CDR3) share the same background color; white background indicates singlets. For the vaccinated control group, only IgM-cell derived sequences are shown. All IgG-cell derived sequences from these individuals can be found in the dataset previously published by Cho et al. (https://doi.org/10.1038/s41586-021-04060-7).

Supplementary Table 4: Sequences, RBD ELISA binding, neutralization, affinity and epitopes of cloned recombinant human anti-SARS-CoV-2 RBD antibodies.

This table summarizes the identity and sequence information of cloned recombinant antibodies derived from RBD-binding memory B cells (as in Supplementary Table 3), as well as their functional characteristics (anti-RBD ELISA binding, neutralization, affinity and competition BLI).

For ELISA anti-RBD binding, half-maximal effective binding concentration (EC50) values below 10000 ng/ml are depicted as acquired; antibodies that show discernible binding above background (isotype control anti-HIV1 antibody 3BNC117) but with EC50s above the level of resolution of our assay are grouped as >10000 ng/ml; antibodies with no discernible binding above background are indicated as "NB". Antibody binding classes (*) are derived from differential competition BLI binding patterns as defined in Fig. 3m, n and Ext. Data Fig. 6. For the vaccinated control group, only IgM-cell derived antibodies are shown. All IgG sequence-derived recombinantly expressed antibodies from these individuals can be found in the dataset previously published by Cho et al. (https://doi.org/10.1038/s41586-021-04060-7).

Supplementary Table 5: Sequences of mouse germinal center (GC) B cell antibodies.
This table summarizes the antibody sequences of single FACS-sorted GC B cells isolated from mouse popliteal lymph nodes 11 days after foot pad immunization with recombinant RBD (as shown in Fig. 4a and Ext. Data Fig. 7a). Sequences derived from individual mice (M1 to M12) are in their respective sheets, as indicated. Clonally related sequences (same V and J genes for both heavy and light chains, and similar CDR3) share the same background color; white background indicates singlets. Binding (y) vs. non-binding (n) by flow cytometry status denotes whether the respective transcript originates from a cell that fell into the RBD-binding gate (Ext. Data Fig. 7b) or not, as assessed by index sorting. Accordingly, a clone is defined as binding (Fig. 4d-e) if one or more member sequences are binding.

Supplementary Table 6: Sequences and binding affinities of cloned recombinant monoclonal mouse GC Fabs.
This table summarizes the identity and sequence information, and monovalent binding affinities of cloned recombinant antibodies (Fabs) derived from mouse GC B cells (as in Supplementary Table 5). Monovalent Fab binding to RBD was assessed by BLI (see methods). Fabs that did not reach saturation when captured on the biosensor (loading phase) at the concentration tested are indicated with asterisks (*). These Fabs could not be assessed for antigen binding and are therefore indicated as "N/D" (not determined). Affinity constants (Kd) are shown for binding antibodies whereas non-binding antibodies are indicated with NB.
