# Supplementary material for: Antibody feedback regulates immune memory after SARS-CoV-2 mRNA vaccination
Source: Nature. 2022 Dec 6;613(7945):735–42. doi: 10.1038/s41586-022-05609-w (PMC9876794; doi:10.1038/s41586-022-05609-w)
Supplement: Supplementary file 2 — Reporting Summary [file 41586_2022_5609_MOESM2_ESM.pdf]

nature portfolio

Corresponding author(s): Theodora Hatziioannou, Paul Bieniasz, Marina Caskey, Michel C. Nussenzweig

Last updated by author(s): Nov 17, 2022

Reporting Summary

Nature Portfolio wishes to improve the reproducibility of the work that we publish. This form provides structure for consistency and transparency in reporting. For further information on Nature Portfolio policies, see our Editorial Policies and the Editorial Policy Checklist.

Statistics

For all statistical analyses, confirm that the following items are present in the figure legend, table legend, main text, or Methods section.

n/a	Confirmed	
☐	☒	The exact sample size (*n*) for each experimental group/condition, given as a discrete number and unit of measurement
☐	☒	A statement on whether measurements were taken from distinct samples or whether the same sample was measured repeatedly
☐	☒	The statistical test(s) used AND whether they are one- or two-sided<br>*Only common tests should be described solely by name; describe more complex techniques in the Methods section.*
☒	☐	A description of all covariates tested
☐	☒	A description of any assumptions or corrections, such as tests of normality and adjustment for multiple comparisons
☐	☒	A full description of the statistical parameters including central tendency (e.g. means) or other basic estimates (e.g. regression coefficient) AND variation (e.g. standard deviation) or associated estimates of uncertainty (e.g. confidence intervals)
☐	☒	For null hypothesis testing, the test statistic (e.g. *F*, *t*, *r*) with confidence intervals, effect sizes, degrees of freedom and *P* value noted<br>*Give P values as exact values whenever suitable.*
☒	☐	For Bayesian analysis, information on the choice of priors and Markov chain Monte Carlo settings
☒	☐	For hierarchical and complex designs, identification of the appropriate level for tests and full reporting of outcomes
☐	☒	Estimates of effect sizes (e.g. Cohen's *d*, Pearson's *r*), indicating how they were calculated

Our web collection on statistics for biologists contains articles on many of the points above.

Software and code

Policy information about availability of computer code

Data collection	IRIS by iMedRIS version 11.01 for clinical data collection and management; BD FACSDiva Software Version 8.0.2 for flow sorting; ClarioStar Multimode reader by BMG Labtech (software version 5.70.R3) for neutralization assays; Omega 5.11 by BMG Labtech was used for ELISA Assays; Forte Bio Octet Data Acquisition software (version 11.1.3.25) for biolayer interferometry (BLI).
Data analysis	FlowJo 10.6.2 for FACS analysis; GraphPad Prism 9.3; Microsoft Excel 16.5.7; MacVector 17.5.4 for sequence analysis; Omega MARS V2.10 by BMG Labtech for luminometer/ELISA; Adobe Illustrator 2022; Geneious Prime (Versions 2020.1.2 and 2022.1.1); BBDuk (v38.93) for sequencing read processing, scripts and the data used to process antibody sequences are available on GitHub (https://github.com/stratust/igpipeline); Forte Bio Data Analysis HT (version 11.1.3.50) for BLI curve fitting.

For manuscripts utilizing custom algorithms or software that are central to the research but not yet described in published literature, software must be made available to editors and reviewers. We strongly encourage code deposition in a community repository (e.g. GitHub). See the Nature Portfolio guidelines for submitting code & software for further information.

Data

Policy information about availability of data

All manuscripts must include a data availability statement. This statement should provide the following information, where applicable:

- Accession codes, unique identifiers, or web links for publicly available datasets
- A description of any restrictions on data availability
- For clinical datasets or third party data, please ensure that the statement adheres to our policy

Data are provided in Supplementary Tables 1-6. The raw sequencing data associated with Figs. 2 and 4 have been deposited at Github (https://github.com/stratust/igpipeline/tree/igpipeline2_timepoint_v2). This study also uses data from “A Public Database of Memory and Naive B-Cell Receptor Sequences” (https://

doi.org/10.5061/dryad.35ks2), PDB (6VYB and 6NB6), cAb-Rep (https://cab-rep.c2b2.columbia.edu/), Sequence Read Archive (accession SRP010970), and from "High frequency of shared clonotypes in human B cell receptor repertoires" (https://doi.org/10.1038/s41586-019-0934-8).

Field-specific reporting

Please select the one below that is the best fit for your research. If you are not sure, read the appropriate sections before making your selection.

☒ Life sciences ☐ Behavioural & social sciences ☐ Ecological, evolutionary & environmental sciences

For a reference copy of the document with all sections, see nature.com/documents/nr-reporting-summary-flat.pdf

Life sciences study design

All studies must disclose on these points even when the disclosure is negative.

Sample size	No a priori sample size calculations were performed. The sample size of 18 individuals (mAb recipients) derives from practical reasons in that it is purely based on how many study participants of the phase 1 study (NCT04700163) elected to subsequently receive mRNA vaccination, remained SARS-CoV-2 infection-naive throughout the study observation period, and could be recruited for serial blood donations at the Rockefeller University Hospital in New York City. Individuals from the vaccinated controls (n=31) were not de novo recruited and have previously been reported on extensively (Cho et al., 2021 and Muecksch et al., 2022). Previous studies, such as the aforementioned Cho et al. and Muecksch et al. have also shown that a sample size of 10-30 individuals can yield representative biological insights as pertains to plasma antibody measurements and detailed molecular assays of memory B cells, thereby empirically validating our sample size selection. For further details about the human study subjects see Supplementary Tables 1 and 2.<br><br>For mouse experiments (related to Fig. 4 and Ext. Data Fig. 7), the sample size of 6 individual animals per group was also not predetermined by statistical sample size calculations. Rather, it corresponds to a sample size that is generally accepted in the field, as it allows for rigorous hypothesis testing, simultaneously keeping the number of animals as small as possible while still being able to meet the scientific objectives (as per the 3R and ARRIVE guidelines).
Data exclusions	No data were excluded from the analysis.
Replication	All experiments successfully performed at least twice.
Randomization	This is not relevant as this is an observational study.
Blinding	This is not relevant as this is an observational study.

Reporting for specific materials, systems and methods

We require information from authors about some types of materials, experimental systems and methods used in many studies. Here, indicate whether each material, system or method listed is relevant to your study. If you are not sure if a list item applies to your research, read the appropriate section before selecting a response.

Materials & experimental systems

n/a	Involved in the study	
☐	☒	Antibodies
☐	☒	Eukaryotic cell lines
☒	☐	Palaeontology and archaeology
☐	☒	Animals and other organisms
☐	☒	Human research participants
☐	☒	Clinical data
☒	☐	Dual use research of concern

Methods

n/a	Involved in the study	
☒	☐	ChIP-seq
☐	☒	Flow cytometry
☒	☐	MRI-based neuroimaging

Antibodies

Antibodies used

1. Mouse anti-human CD20-PECy7 (BD Biosciences, 335793), clone L27
2. Mouse anti-human CD3-APC-eFluro 780 (Invitrogen, 47-0037-41), clone OKT3
3. Mouse anti-human CD8-APC-421eFluro 780 (Invitrogen, 47-0086-42), clone OKT8
4. Mouse anti-human CD16-APC-eFluro 780 (Invitrogen, 47-0168-41), clone eBioCB16
5. Mouse anti-human CD14-APC-eFluro 780 (Invitrogen, 47-0149-4), clone 61D3
6. Zombie NIR (BioLegend, 423105)
7. Mouse anti-human CD19-BV605 (Biolegend, 302244), clone HIB19
8. Mouse anti-human IgG-PECF594 (BD Bioscience, 562538), clone G18-145
9. Mouse anti-human IgM-AF700 (Biolegend, 314538), clone MHM-88
10. Peroxidase Goat anti-Human IgG Jackson Immuno Research 109-036-088
11. Peroxidase Goat anti-Human IgM Jackson Immuno Research 109-035-129

12. Rat anti-mouse T and -B cell activation antigen-FITC (BD Biosciences, 553666), clone GL7
13. Rat anti-CD38-PB (Biolegend, 102720), clone 90
14. Rat anti-mouse/human CD45R/B220-BV605 (Biolegend, 103244), clone RA3-6B2
15. Rat anti-mouse CD4-APC-eFluor780 (Invitrogen, 47-0042-82), clone RM4-5
16. Rat anti-mouse CD8a-APC-eFluor780 (Invitrogen, 47-0081-82), clone 53-6.7
17. Anti-mouse NK1.1-APC-eFluor780 (Invitrogen, 47-5941-82), clone PK136
18. Rat anti-mouse F4/80-APC-eFluor780 (Invitrogen, 47-4801-82), clone BM8
19. Armenian hamster anti-mouse CD95-PE-Cy7 (BD Biosciences, 557653), clone Jo2
20. Mouse anti-human CD38-BV421 (Biolegend, 303526), clone HIT2

Validation

All antibodies are commercially available and validated by manufacturers. Additional information can be found on the respective product websites listed below:
1. https://www.bdbiosciences.com/en-us/products/reagents/flow-cytometry-reagents/clinical-discovery-research/single-color-antibodies-ruo-gmp/pe-cy-7-mouse-anti-human-cd20.335793
2. https://https://www.biolegend.com/en-us/products/zombie-nir-fixable-viability-kit-8657www.thermofisher.com/antibody/product/CD3-Antibody-clone-OKT3-Monoclonal/47-0037-42
3. https://www.thermofisher.com/antibody/product/CD8a-Antibody-clone-OKT8-OKT-8-Monoclonal/47-0086-42
4. https://www.thermofisher.com/antibody/product/CD16-Antibody-clone-eBioCB16-CB16-Monoclonal/47-0168-42
5. https://www.thermofisher.com/antibody/product/CD14-Antibody-clone-61D3-Monoclonal/47-0149-42
6. https://www.biolegend.com/en-us/products/zombie-nir-fixable-viability-kit-8657
7. https://www.biolegend.com/en-us/products/brilliant-violet-605-anti-human-cd19-antibody-8483?GroupID=BLG5913
8. https://www.bdbiosciences.com/en-us/products/reagents/flow-cytometry-reagents/research-reagents/single-color-antibodies-ruo/pe-cf594-mouse-anti-human-igg.562538
9. https://www.biolegend.com/fr-lu/products/alexa-fluor-700-anti-human-igm-antibody-12507
10. https://www.jacksonimmuno.com/catalog/products/109-036-088
11. https://www.jacksonimmuno.com/catalog/products/109-035-129
12. https://www.bdbiosciences.com/en-us/products/reagents/flow-cytometry-reagents/research-reagents/single-color-antibodies-ruo/fitc-rat-anti-mouse-t-and-b-cell-activation-antigen.553666
13. https://www.biolegend.com/de-at/products/pacific-blue-anti-mouse-cd38-antibody-6652
14. https://www.biolegend.com/fr-fr/products/brilliant-violet-605-anti-mouse-human-cd45r-b220-antibody-7870
15. https://www.thermofisher.com/antibody/product/CD4-Antibody-clone-RM4-5-Monoclonal/47-0042-82
16. https://www.thermofisher.com/antibody/product/CD8a-Antibody-clone-53-6-7-Monoclonal/47-0081-82
17. https://www.thermofisher.com/antibody/product/NK1-1-Antibody-clone-PK136-Monoclonal/47-5941-82
18. https://www.thermofisher.com/antibody/product/F4-80-Antibody-clone-BM8-Monoclonal/47-4801-82
19. https://www.bdbiosciences.com/en-us/products/reagents/flow-cytometry-reagents/research-reagents/single-color-antibodies-ruo/pe-cy-7-hamster-anti-mouse-cd95.557653
20. https://www.biolegend.com/it-it/products/brilliant-violet-421-anti-human-cd38-antibody-7145

Eukaryotic cell lines

Policy information about cell lines

Cell line source(s)

293T (ATCC CRL-11268)
293T/ACE2* (generated in-house for Robbiani, D. et al. Nature 584, doi.org/10.1038/s41586-020-2456-9, and maintained since)
HT1080/ACE2.cl14 (generated in-house for Schmidt, F. et al. J Exp Med 217, doi:10.1084/jem.20201181, and maintained since)
Expi293F (GIBCO/Thermo Fisher, A14527)

Authentication

Not authenticated after purchase from ATCC and GIBCO/Thermo Fisher, respectively.

Mycoplasma contamination

All cell lines tested negative for mycoplasma contamination by Hoechst staining.

Commonly misidentified lines
(See ICLAC register)

No commonly misidentified cell lines were used.

Animals and other organisms

Policy information about studies involving animals; ARRIVE guidelines recommended for reporting animal research

Laboratory animals

C57BL/6 mice purchased from Jackson laboratory were used. All mice used were females between 6-12 weeks of age. Mice were housed at a temperature of 72 °F and humidity of 30–70% in a 12-h light/dark cycle with ad libitum access to food and water.

Wild animals

no usage of wild animals

Field-collected samples

none

Ethics oversight

All animal procedures and experiments were performed according to protocols approved by the Rockefeller University Institutional Animal Care and Use Committee (IACUC).

Note that full information on the approval of the study protocol must also be provided in the manuscript.

Human research participants

Policy information about studies involving human research participants

Population characteristics	Participants in the monoclonal recipient group were healthy volunteers who had previously received a single dose of a combination of C144-LS and C135-LS, two human IgG1 neutralizing anti-RBD monoclonal antibodies (first characterized in Robbiani et al., 2020), in a phase 1, first-in-humans study to assess the safety and tolerability as well as the pharmacokinetics of the two antibodies (NCT04700163), and who subsequently got vaccinated with the initial two-dose regimen of either the Moderna (mRNA-1273) or Pfizer-BioNTech (BNT162b2) mRNA vaccines against the wildtype (Wuhan-Hu-1) strain of the severe acute respiratory syndrome coronavirus 2 (SARS-CoV-2). Of note, vaccinations were at the discretion of each individual participant and their health care providers and not part of our study design, which was purely observational in nature. Particpants were 43 (24-64) years old (median (range)), 5 out of 18 participants were female. 6 participants received the Moderna (mRNA-1273) and 12 received the Pfizer-BioNTech (BNT162b2) vaccine. Participants in the vaccinated controls group were not de novo recruited for this study and we defer to Supplementary Information Tables S1 and S2, as well as Cho et al., 2021 and Muecksch et al., 2022 for more details.
Recruitment	Recruitment of individuals into the antibody recipient group of this study was pragmatic, in that all eligible participants (no history of SARS-CoV-2 infection, having received active agent C135-LS and C144-LS and not placebo, subsequent vaccination with 2 doses of either the Moderna (Spikevax, mRNA-1273) or Pfizer-BioNTech (Comirnaty, BNT162b2) mRNA vaccines against the wildtype (Wuhan-Hu-1) strain) of the phase 1 clinical trial (NCT04700163) were offered enrollment in the observational study reported on herein. As with all human subjects research based on healthy volunteers, the study cohort composition may be biased toward individuals with more access to health- and science-related resources. However, due to the direct linkage of this observational study to the phase 1 trial (NCT04700163), additional biases, such as self-selection bias, are unlikely.<br><br>No further recruitment efforts were undertaken, as the control group was not de novo recruited for this study. A detailed description of their recruitment can be found in Cho et al., 2021 and Muecksch et al., 2022.
Ethics oversight	The study was performed in compliance with all relevant ethical regulations and the protocols (CGA-1015 and DRO-1006) for studies with human participants were approved by the Institutional Review Board of the Rockefeller University.

Note that full information on the approval of the study protocol must also be provided in the manuscript.

Clinical data

Policy information about clinical studies

All manuscripts should comply with the ICMJE guidelines for publication of clinical research and a completed CONSORT checklist must be included with all submissions.

Clinical trial registration	NCT04700163
Study protocol	The study protocol can be accessed under clinicaltrials.gov (https://clinicaltrials.gov/ct2/show/NCT04700163)
Data collection	The study "A Phase 1, Open Label, Dose-escalation Study of the Safety and Pharmacokinetics of a Combination of Two Anti-SARS-CoV-2 mAbs (C144-LS and C135-LS) in Healthy Volunteers" (NCT04700163) was conducted at The Rockefeller University between January 11, 2021 and February 2, 2022.
Outcomes	NCT04700163 was conducted to assess the safety and tolerability, as well as the pharmacokinetics of C144-LS and C135-LS, with adverse events and phamacokinetic properties of the infused antibodies as its primary and secondary outcomes.<br><br>However, the study presented here explicitly does not report on the pre-defined endpoints of NCT04700163. Instead, the data presented in this manuscript merely represents an observational study of the immune response to vaccination in participants of NCT04700163, which does not constitute a pre-specified outcome of NCT04700163.

Flow Cytometry

Plots

Confirm that:

☒ The axis labels state the marker and fluorochrome used (e.g. CD4-FITC).

☒ The axis scales are clearly visible. Include numbers along axes only for bottom left plot of group (a 'group' is an analysis of identical markers).

☒ All plots are contour plots with outliers or pseudocolor plots.

☒ A numerical value for number of cells or percentage (with statistics) is provided.

Methodology

Sample preparation	For human samples, whole blood samples were obtained from study participants recruited through Rockefeller University Hospital. Peripheral blood mononuclear cells (PBMCs) were separated by Ficoll gradient centrifugation. Prior to sorting, PBMCs were enriched for B cells using a Miltenyi Biotech pan B cell isolation kit (cat. no. 130-101-638) and LS columns (cat. no. 130-042-401).

For mouse experiments, popliteal lymph nodes from mice 11 days after immunization were isolated and collected in FACS buffer (1x PBS, 2% FBS, 2 mM EDTA). Single cell suspensions of the pooled popliteal lymph node samples from each respective mouse were subsequently processed as described.

Instrument	FACS Aria III (Becton Dickinson), BD FACSymphony S6 (Becton Dickinson)
Software	BD FACSDiva Software Version 8.0.2 and FlowJo 10.6.2
Cell population abundance	For the human experiments, sorting efficiency ranged from 40% to 80%. This is calculated based on the number of antibody sequences that could be successfully PCR-amplified from single-sorted cells from each donor using either IgM or IgG heavy chain-specific primers (see Robbiani et al., 2020 and Wang et al., 2020) in conjunction with IgK and IgL-specific light chain primers.<br><br>For the mouse experiments, GC B cell abundance was not a limiting factor for cell sorting. Sorting efficiencies (based on the same calculation as above) were slightly lower (between 30 to 80%), with the notable difference that only IgK-specific light chain primers were used.
Gating strategy	For human experiments, cells were first gated for single cells in FSC-A versus FSC-H, and then for lymphocytes in FSC-A (x-axis) versus SSC-A (y-axis). We then selected for either CD20+ (cell sorting) or CD19+CD20+ (flow-cytometric phenotyping) and Dump- B Cells in dump (anti-CD3-eFluro 780, anti-CD16-eFluro 780, anti-CD8-eFluro 780, anti-CD14-eFluro 780, Zombie NIR) versus CD20 (anti-CD20-PE-Cy7) or versus CD19 (anti-CD19-BV605); dump-negative was considered to be signal less than 1200, CD19-positive was taken to be signal greater than 1000, and CD20-positive was taken to be signal greater than 500. We then gated for Ova- B cells in CD20 versus Ova-BV711; Ova-negative was considered to be all cells with signal less than 1200 (flow-cytometry) or 300 (cell sorting). We selected for Sars-CoV-2 RBD double-positive cells in RBD-PE versus RBD-AlexaFluor 647; this gate was made along the 45° diagonal, above 1000 (flow-cytometry) or 500 (cell-sorting) on both axes. IgG+ (IgG-PECF594) versus IgM+ (IgM-AF700) cells among RBD dual-labelled cells were gated using mutually exclusive gates with signals above 1000 for each.<br><br>For mouse experiments, gating was as detailed in Ext. Data Fig. 7a. Briefly, single live cells were gated to only include cells negative for staining with anti-CD4-APC-eFluor780, anti-CD8a-APC-eFluor780, anti-NK1.1-APC-eFluor780, anti-F4/80-APC-eFluor780 and and Zombie NIR to exclude dead and irrelevant cell populations. Next, cells positive for staining with anti-CD45R/B220-BV605 were considered B cells. B cells with MFIs <1000 for staining with anti-CD38-PB/BV421 and positive for staining with anti-GL7-FITC and anti-CD95-PE-Cy7 were considered GC B cells. Among those, cells with MFIs higher than 500 for RBD-A647 and 1000 for RBD-PE were deemed RBD-binding. Cell sorting was done on cells in the GC B cell gate agnostic of binding to RBD. RBD-binding status of single-sorted cells was established post-factum through index sorting data, using the same gating as in Ext. Data Fig 7a and b.

☒ Tick this box to confirm that a figure exemplifying the gating strategy is provided in the Supplementary Information.
